# Extracellular Vesicles from Mesenchymal Stromal Cells for the Treatment of Inflammation-Related Conditions

**DOI:** 10.3390/ijms22063023

**Published:** 2021-03-16

**Authors:** Sean T. Ryan, Elham Hosseini-Beheshti, Dinara Afrose, Xianting Ding, Binbin Xia, Georges E. Grau, Christopher B. Little, Lana McClements, Jiao Jiao Li

**Affiliations:** 1 Kolling Institute, Faculty of Medicine and Health, University of Sydney, St. Leonards, NSW 2065, Australia; sean@ryans.com.au (S.T.R.); christopher.little@sydney.edu.au (C.B.L.); 2 Faculty of Medicine and Health, School of Medical Sciences, University of Sydney, Camperdown, NSW 2006, Australia; elham.beheshti@sydney.edu.au (E.H.-B.); georges.grau@sydney.edu.au (G.E.G.); 3 Faculty of Science, School of Life Sciences, University of Technology Sydney, Ultimo, NSW 2007, Australia; MstDinara.Afrose@student.uts.edu.au (D.A.); lana.mcclements@uts.edu.au (L.M.); 4 Institute for Personalized Medicine, School of Biomedical Engineering, Shanghai Jiao Tong University, Shanghai 200030, China; dingxianting@sjtu.edu.cn; 5 Faculty of Engineering and IT, School of Biomedical Engineering, University of Technology Sydney, Ultimo, NSW 2007, Australia; binbin.xia@uts.edu.au

**Keywords:** mesenchymal stromal cells, extracellular vesicles, inflammation, regeneration, osteoarthritis, rheumatoid arthritis, Alzheimer’s disease, cardiovascular disease, preeclampsia

## Abstract

Over the past two decades, mesenchymal stromal cells (MSCs) have demonstrated great potential in the treatment of inflammation-related conditions. Numerous early stage clinical trials have suggested that this treatment strategy has potential to lead to significant improvements in clinical outcomes. While promising, there remain substantial regulatory hurdles, safety concerns, and logistical issues that need to be addressed before cell-based treatments can have widespread clinical impact. These drawbacks, along with research aimed at elucidating the mechanisms by which MSCs exert their therapeutic effects, have inspired the development of extracellular vesicles (EVs) as anti-inflammatory therapeutic agents. The use of MSC-derived EVs for treating inflammation-related conditions has shown therapeutic potential in both in vitro and small animal studies. This review will explore the current research landscape pertaining to the use of MSC-derived EVs as anti-inflammatory and pro-regenerative agents in a range of inflammation-related conditions: osteoarthritis, rheumatoid arthritis, Alzheimer’s disease, cardiovascular disease, and preeclampsia. Along with this, the mechanisms by which MSC-derived EVs exert their beneficial effects on the damaged or degenerative tissues will be reviewed, giving insight into their therapeutic potential. Challenges and future perspectives on the use of MSC-derived EVs for the treatment of inflammation-related conditions will be discussed.

## 1. Introduction

Inflammation is a crucial mechanism initiated by the body as a first line of defence against harmful stimuli such as pathogens, tissue damage, radiation, and toxic compounds [1]. An acute inflammatory response is normally triggered by immune cells sensing a pathogen or endogenous stress signal, resulting in the release of pro-inflammatory cytokines and chemokines. This reaction can have a multitude of effects, including neutrophil and macrophage activation, vasodilation, hypotension, induction of capillary leakage, and platelet activation [1,2]. These effects typically facilitate tissue regeneration or the clearance of infection, ultimately leading to the removal of the initial harmful stimuli. Once cleared of harmful stimuli, the multifaceted process of inflammation resolution can begin, which involves substantial reprogramming of cells to the anti-inflammatory phenotype [2]. Unfortunately, acute inflammation can often progress into chronic non-resolving inflammation, which may elicit more harm to the body than the initial stimuli that triggered the inflammatory response [3]. Though not the primary cause, non-resolving chronic inflammation has been identified to play an important role in the pathogenesis of a myriad of debilitating diseases including rheumatoid arthritis [2,4], atherosclerosis [2,5], Alzheimer’s disease [6], various cancers [2,7,8,9], asthma [10], type 2 diabetes [11], diabetic nephropathy [12], osteoarthritis [13,14,15], multiple sclerosis [16], depression [17], chronic rhinosinusitis [18], idiopathic pulmonary fibrosis [19], and atrial fibrillation [20]. These diseases share many common pathophysiological mechanisms, including the activation of inflammatory cells, release of soluble inflammatory factors (most notably cytokines and chemokines), and extracellular matrix (ECM) remodelling [21].

With such a long list of conditions in which non-resolving inflammation plays a key role, there is no doubt that it imposes an immense burden on society. Unfortunately, commonly used anti-inflammatory treatments such as non-steroidal anti-inflammatory drugs (NSAIDs) and glucocorticoids appear to merely relieve symptoms of the underlying disease, and there is little evidence to demonstrate that these treatments have any effectiveness in ceasing disease progression [22]. As such, there is an urgent need to develop new therapeutic strategies, which perhaps can act on multiple pathways of disease progression rather than only targeting the inflammatory characteristics.

Mesenchymal stromal cells (MSCs), previously commonly referred to as mesenchymal stem cells [23], are the most widely explored cell type for cell-based therapeutics, and their use in clinical trials to treat a wide range of diseases has increased dramatically over the past two decades [24]. The literature provides ample evidence of studies showing the beneficial effects of MSCs when applied for treating inflammatory diseases in animal models [25,26], with evidence in multiple tissue types including cardiovascular (myocardial infarction, vascular disease, peripheral artery disease, preeclampsia); neural (multiple sclerosis, Parkinson’s disease, Alzheimer’s disease); and osteochondral (rheumatoid arthritis, osteoarthritis) [26,27,28,29]. As such, there is an ongoing urge within the scientific community to translate these promising findings to humans. It was initially believed that the therapeutic potential of MSCs was a function of injected MSCs engrafting to existing cellular structures, and subsequently differentiating and facilitating the formation of neo-tissue [30]. However, this belief has been subverted in recent years. It has been widely observed that implanted MSCs show very low levels of engraftment (less than 3%) in the target tissue [31], with the vast majority of the population of implanted cells being rapidly cleared [32]. For this reason, other mechanisms have been investigated, and it is now evident that the regenerative, anti-inflammatory, and immunomodulatory capacity of MSCs is exerted through their secretion of paracrine factors [33,34,35].

The MSC secretome accounts for all molecules secreted by the cell. It includes a variety of chemokines, cytokines, immunomodulatory factors, and ECM components, along with a range of other proteins, nucleic acids, and lipids [32]. It is suggested that once MSCs are implanted into damaged or diseased tissue, they secrete a host of anti-inflammatory and regenerative factors that elicit a therapeutic response. Importantly, the secretion profile appears to be a function of the microenvironment around the secreting cell, for instance, MSCs exposed to inflammatory signals can elicit an enhanced secretory profile [36]. However, the majority of investigations surrounding this observation have been in vitro gene expression or proteomic studies and require further in vivo validation [32].

It has been suggested that the apoptosis or phagocytosis of implanted MSCs act as the trigger for the observed immunomodulatory effects elicited by MSCs [32]. There are so far two key observations supporting this mechanism. First, observations in mouse models of graft-versus-host disease have demonstrated that, for MSCs to exert their immunosuppressive effects, they must first undergo natural killer cell/T-cell induced apoptosis [37]. Second, observations in a mouse model showed that injected populations of MSCs were rapidly cleared through monocytic phagocytosis. The monocytes that phagocytosed the MSCs were shown to modulate their phenotype, which changed the course of the immune response [38]. These two observations provide a potential hypothesis for the mechanisms of MSC-mediated immunomodulation, though further studies are required to confirm the details.

Aside from the above two proposed mechanisms underlying the therapeutic effects of MSCs, a third mechanism has gained increasing attention in recent years: extracellular vesicles (EVs) derived from MSCs. This will be the topic of focus in this review that will be discussed in the context of treating inflammation-related conditions.

## 2. The Fundamentals of Extracellular Vesicles

EV is an umbrella definition which encompasses all vesicles released or ’shed’ by cells [39]. Typically, EVs have a diameter in the range of 30–2000 nm. They consist of a lipid bi-layer membrane encasing an organelle-free cytosole, which contains a combination of various proteins, lipids, and nucleic acids [40,41]. EVs have been recently discovered as a key mechanism of the intercellular communication network. Since EV release was first observed in rat and sheep reticulocytes in the early 1980s [42,43], an ever-growing number of cells have been shown to release EVs as a form of intercellular communication. Almost all mammalian cell types have demonstrated EV secretion including stem cells, neuronal cells, immune cells, and cancer cells [39,44]. EVs have also been isolated from an extensive range of biological fluids including blood, urine, semen, breast milk, cerebrospinal fluid, bile, amniotic fluid, and ascites fluid [44]. Interestingly, EV secretion has been observed in lower eukaryotes and prokaryotes, with speculations that microbial EVs may mediate the host response to infection [44,45].

**Figure 1:**
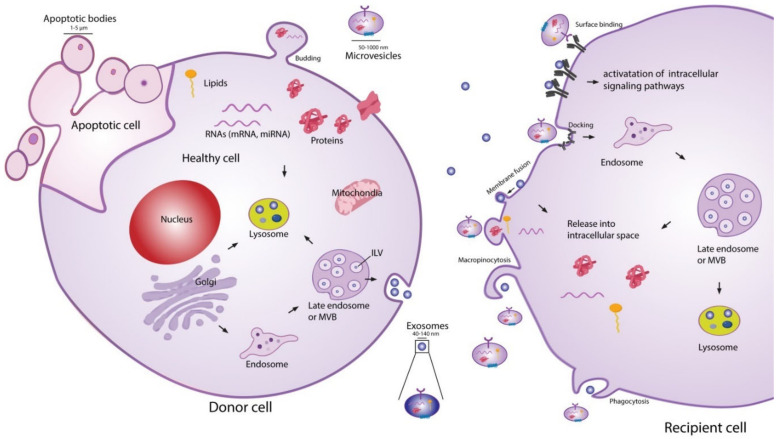
Extracellular vesicle (EV) biogenesis, secretion, and uptake [56]. Exosomes (20–150 nm) are intraluminal vesicles (ILVs) formed by inward budding of the endosomal membrane during maturation of multivesicular body (MVB), which are secreted upon fusion of the MVBs with the plasma membrane. Microvesicles (50–1000 nm) are a heterogeneous group of vesicles with different membranes depending on their origin and morphology. Apoptotic bodies are shedding vesicles derived from apoptotic cells. After their release into the extracellular space, EVs can bind to cell surface receptors to initiate intracellular signalling pathways. EVs can also be internalised through processes such as macropinocytosis and phagocytosis, or by fusion with the plasma membrane. The cargo of EVs consisting of proteins, nucleic acids and lipids are released in the intracellular space or taken up by the endosomal system of the recipient cell. Reproduced with permission from [56].

The exact classification of EVs is still evolving, and the current definition of nomenclature is not consistently used in the literature [46,47]. Presently, EV classifications are based on their size and biogenesis [46], with three widely accepted distinct populations. Exosomes are the most widely studied subpopulation of EVs [48]. Although the size range of exosomes has not been consolidated in the literature, it is generally accepted that they have a diameter in the range of 20–150 nm. The biogenesis of exosomes begins with endocytosis, a process of invagination of the plasma membrane to form an endosome. Within the endocytic pathway, endosomes are classified into three sub-populations: early endosomes, late endosomes, and recycling endosomes [49]. Early endosomes which are not destined for secretion, recycling, or degradation become late endosomes. Late endosomal membrane invagination subsequently forms intraluminal vesicles (ILVs) which contain proteins, lipids, and nucleic acids. At this point, the late endosome now containing a host of small vesicles is deemed a multivesicular body (MVB) [40]. The MVB has two possible routes, either fusing with the lysosome where its contents will be recycled or fusing with the plasma membrane. The latter releases the ILVs into the extracellular space, where they are now referred to as exosomes. This process is visualised in Figure 1. The formation of ILVs is believed to be mainly regulated by two processes. First, the endosomal membrane is enriched for tetraspanins, specifically CD9 and CD63 [50]. Second, the endosomal sorting complexes required for transport (ESCRTs) are present during the process of ILV formation. These two processes regulate the initial inward membrane budding of the late endosome, ILV cargo sorting, and subsequent ILV formation. Although it is generally accepted that the ESCRT pathway is the main mechanism governing exosome formation, there exist supplementary mechanisms of ILV formation such as the syndecan–syntenin–ALIX pathway [40]. Since exosomes arise from endosome membrane invagination, they present common proteins associated with this process across all cell types. These proteins include flotillins, GTPases and annexins (membrane transport and fusion); integrins (adhesion); ALIX and the tetraspanins CD9, CD63, CD81, CD82 (MVB formation); and major histocompatibility complex (MHC) molecules (antigen presentation) [51]. Typically, the lipid composition of exosomes mirrors that of their parent cell. Exosomes are commonly enriched with cholesterol, phosphatidylserine, ceramide, and sphingomyelin [52]. Interestingly, the concentration of diacyl-glycerol and phosphatidyl-choline appear to be lower in exosomes than their parent cells [53]. The nucleic acid content of exosomes typically consists of mRNAs, microRNAs (miRNAs), and other non-coding RNAs [51], although genomic and mitochondrial DNA have also been found in exosomes [54,55].

The second most widely studied subpopulation of EVs are microvesicles (MVs) [48]. It is generally accepted that MVs have a diameter in the range of 50–1000 nm, meaning that they may have a size overlap with exosomes. This creates challenges for purely size-based EV isolation techniques in distinguishing between exosome and MV populations [39]. In contrast to exosomes, MVs are formed through direct shedding from the plasma membrane of the parent cell. The formation of MVs is regulated by aminophospholipid translocases, which control the phospholipid re-distribution in the plasma membrane and the dynamics of cytoskeletal actin-myosin contractions [57]. As MVs form through direct outward budding of the plasma membrane (Figure 1), they share many of the same membrane markers as their parent cell, which may include integrins, selectins, and CD40 ligand [58]. The variations in membrane markers among MVs is a result of the induced changes which occur during the process of nucleation and budding [51]. The cargo carried by MVs, like exosomes, is not simply representative of the cytoplasmic content. Some loading mechanisms such as ARF6 trafficking of proteins and CSE1L nucleic acid export have been identified [59,60]. However, the exact mechanisms of regulation remain incompletely understood and constitute an area of active research. The protein and nucleic acid content of MVs are dependent on the cell type along with the external physiological conditions experienced by the parent cell [40]. A number of proteins are commonly identified in MVs, such as matrix metalloproteinases (MMPs), cytoskeletal components, and glycoproteins [51]. Like exosomes, MVs generally contain a combination of mRNAs, miRNAs, and other non-coding RNAs [51], as well as possible genomic and mitochondrial DNA [54,55].

Apoptotic bodies are the final widely recognised subpopulation of EVs. They are by far the largest in size, ranging 500–5000 nm in diameter, and are produced by outward membrane blebbing on the surface of cells undergoing apoptosis [58,61]. There is no evidence that apoptotic bodies play a role in intercellular communication or have a potential therapeutic effect, although they do show potential to be used as disease biomarkers [62].

EV-cell communication can occur through several distinct pathways: lysis of EVs in the extracellular space releasing their contents, direct EV-cell binding, membrane fusion and release of EV contents, and EV uptake into the endocytic system [56,63]. Ligand-receptor binding associated with EV extracellular content release and direct EV binding are believed to be the mechanisms behind several of the biological effects exerted by EVs on cells, such as growth and angiogenic factor delivery [63]. For the nucleic acids or proteins suspended in the EV cytosol to act as messengers in the recipient cell, the EVs must fuse either with the plasma membrane after ligand-receptor binding, or with the endosomal membrane after endocytosis [63]. Endocytosis of EVs is thought to be the most common route of uptake [40,41,63], although several questions remain to be answered about this uptake route. Since the endocytic pathway inevitably ends with degradation or expulsion from the cell, the cargo carried by the EVs must exit the endosome somehow and find its way into the cytoplasm if it is to alter cell composition and function [40]. Although this phenomenon of endosomal escape has been widely observed, the underlying mechanisms are still unclear [40,64,65]. EV–cell communication is known to be involved in an extensive range of biological processes, including modulation of the immune system [66,67], neuro-biological functions such as synaptic plasticity [68], and stem cell differentiation [69,70].

With the extensive role that EVs play in biological processes, it is unsurprising that they are also heavily involved in the pathogenesis of disease. The most in-depth understanding of this concept is in tumour biology [71]. EVs have been shown to have important roles in promoting tumour cell proliferation [72,73], angiogenesis [73,74], ECM remodelling [75], and metastasis [58,75]. Although beyond the scope of this review, there is a great potential in targeting the phenotype altering mechanisms exerted by EVs in tumour biology to help develop new treatment strategies, as well as to apply stem cell-derived EVs as cancer therapeutics [76]. In the field of regenerative medicine, EVs derived from stem cells are shown to replicate the therapeutic properties of the parent cells, and have demonstrated many beneficial effects such as apoptosis suppression [77], promotion of cellular proliferation [78] and angiogenesis [79], and the ability to modulate the diseased cell phenotype to facilitate tissue regeneration [80]. The precise cargo carried by EVs and the mechanisms which facilitate their regenerative potential are still unclear. However, it is known that EV composition is a function of its cellular origin and physiological conditions [81]. By varying factors such as cellular stress, media composition, and physical stimulation, or by enriching certain miRNAs in the parent cells, it may be possible to optimise the EV composition for specific regenerative applications [82,83,84,85].

**Figure 2:**
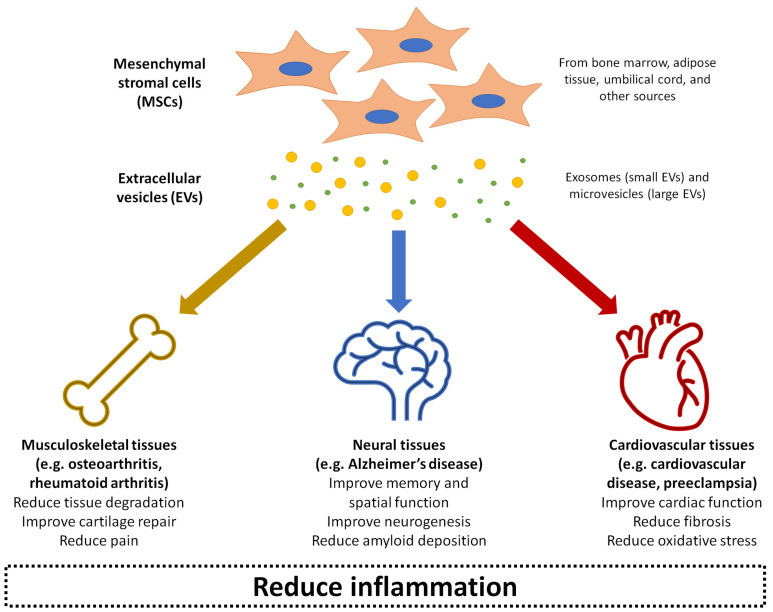
Summary of the application of mesenchymal stromal cell (MSC)-derived EVs in treating inflammation-related conditions as covered in this review: osteoarthritis, rheumatoid arthritis, Alzheimer’s disease, cardiovascular disease, and preeclampsia.

Over the past decade, MSC-derived EVs have been increasingly explored in regenerative medicine to treat disease or promote repair through local delivery in a range of tissue types, including cardiovascular, musculoskeletal, neural, renal, hepatic, lung, dermal, and reproductive tissues [56,86]. It is thought that the MSC-derived EVs can deliver the same anti-inflammatory and trophic effects as the parent cells [87]. Compared to injecting live cells into tissues, MSC-derived EVs bypass potential safety concerns of the MSCs exhibiting uncontrollable behaviour or differentiating into problematic tissue at the site of injection [88]. The EVs also have an additional advantage of presenting minimal toxicity and immunogenicity, even when applied xenogenetically as a large dose at high frequency [89]. The rest of this review will summarise the current state of research into MSC-derived EVs as therapeutic agents for treating a number of inflammation-related conditions: osteoarthritis, rheumatoid arthritis, Alzheimer’s disease, cardiovascular disease, and preeclampsia (Figure 2). For each of these conditions, evidence related to the therapeutic effects of MSC-derived EVs has been collected from a range of experimental studies published within the last ten years, as shown in Table 1. These conditions represent examples of diseases with significant societal impact, where pathogenesis is closely linked with inflammation in musculoskeletal, neural, and cardiovascular tissues as three major body systems. MSC-derived EVs have also demonstrated beneficial effects in other conditions and body systems impacted by inflammation, such as graft-versus-host disease [90], kidney disease [91], liver failure [92], and skin wounds [93], although a detailed discussion of these is beyond the scope of this review.

## 3. Extracellular Vesicles from Mesenchymal Stem Cells for the Treatment of Osteoarthritis

Osteoarthritis (OA) is the most prevalent joint disease globally, affecting 18% of women and 10% of men over the age of 60 [94]. While OA is generally characterised by the degeneration of articular cartilage, it is a disease affecting the entire joint including the subchondral bone and synovium [95]. Although not the primary defining feature of OA, chronic inflammation forms an important part of the catabolic environment that induces the irreversible progression of joint degeneration [96]. The exact pathogenesis of OA is incompletely understood, but it is generally accepted that antagonistic biomechanics acting on a vulnerable joint is intrinsically linked to disease progression [94]. A number of risk factors including age, obesity, abnormal joint morphology, and prior joint injury are strongly associated with the development of OA. Once the disease progresses, the regeneration of damaged joint structures is unlikely. Non-surgical treatments such as anti-inflammatory medication and intra-articular injections of corticosteroids or hyaluronic acid may help to relieve pain, although these have shown little to no benefit in slowing disease progression [97]. With a lack of viable treatment options, the final destination for most patients after all options have been exhausted is total joint replacement, which surgically removes the diseased joint. Although this can lead to significant pain reduction and overall improved quality of life, the level of activity post-surgery is relatively low compared to pre-replacement levels due to component failure or loosening [98], and the implant may need revision in younger patients due to having a limited lifetime of approximately 20 years [99].

The use of MSCs to treat OA has shown promise over the last decade, with numerous early clinical studies suggesting that this approach is safe and effective, and may lead to significant improvements in clinical outcomes along with some preservation or regeneration of damaged joint tissues [100,101]. However, there remain significant hurdles before MSCs can be scaled up for widespread clinical use, due to limited cell survival following injection, inability to be used as an ‘off-the-shelf’ therapy, and regulatory issues associated with the injection of live cells [102]. The use of MSC-derived EVs circumvents these issues, and have shown promising preliminary outcomes in both in vitro and small animal models of OA, as described below and in recent reviews on this topic [87,103].

The 16 studies on OA described in Table 1 demonstrate relatively consistent therapeutic effects of MSC-derived EVs. The MSCs used to generate EVs were derived from many different sources, including bone marrow [83,104,105,106,107], adipose tissue [82,85,108,109], synovial membrane [84,110], embryonic stem cells [111,112,113], and induced pluripotent stem cells [110,114]. The majority of studies used exosomes [83,84,85,105,106,110,111,112,113,114,115], while others used MVs [107] or a heterogenous population of EVs that likely contained both exosomes and MVs [82,108,109]. One study also compared the effects of exosomes and MVs [104]. There was not a consensus among the studies on the methods of identifying EV populations, with some purely based on size and others based on size and protein markers, although the size and exact protein markers were also not consistent. The terminology used to refer to EV populations varied among studies, with MVs and microparticles being used interchangeably.

The in vitro experiments conducted using MSC-derived EVs in OA cell models showed that the EVs were quickly internalised by the treated cells, usually within 30 min [82,84,106,107]. It was also widely observed that EVs improved the migration and proliferation potential of the treated cells [83,84,110,113,114,115], together with increased viability and reduced rate of apoptosis, and that the improvements were dose-dependent [85,104,107,113,114,115]. EV treatment of OA chondrocytes and fibroblasts commonly resulted in the upregulation of anabolic proteins such as aggrecan, and collagen types I and II [85,104,106,109,111]. This was accompanied by the downregulation of catabolic markers such as MMP-13 and ADAMTS5 [83,85,109,115]. EV treatment also showed anti-immunomodulatory and anti-inflammatory effects, through the suppression of COX-2, IL-1α, IL-1β, IL-6, IL-8, IL-17, and TNF-α [106,109], as well as the inhibition of macrophage activation [113].

Among the 15 studies that performed in vivo investigations, six were performed in rats [84,105,109,112,113,115], seven in mice [82,83,85,104,109,110,111], and two in rabbits [107,114]. The method of inducing OA differed among studies: injection of monosodium iodoacetate (MIA) [109] or collagenase [83,104,110,115]), surgical destabilisation of the medial meniscus (DMM) [85,109,111] or medical collateral ligament and medial meniscus transection [84], induction of osteochondral defects [107,112,113,114], and muscular injury through cardiotoxin injection [82]. All studies demonstrated a positive therapeutic effect exerted following EV administration.

In MIA and collagenase-induced OA models, all studies showed that EVs could attenuate OA progression to varying degrees [83,84,85,105,109,110,111,115], with some studies demonstrating the regeneration of osteochondral tissue [83,84,104,105,110,115]. Many reported a suppression of catabolic markers (MMP-13, ADAMTS5) [85,105,109] and an increase in anabolic markers (collagen type II, proteoglycan, aggrecan) [85,105,111]. Other reported effects of EVs included significant analgesia [105], subchondral bone regeneration [105], and reduced synovial inflammation [109]. EVs enriched with specific miRNAs (IncRNA KLF3- AS1, miR92a-3p, miR-140-5p, miR1005p) were shown to produce improved therapeutic outcomes [83,84,85,115].

Similarly, in surgically induced OA models, all studies showed that EVs could attenuate OA progression [84,85,109,111], together with downregulation of catabolic markers (MMP-13, ADAMTS5) and upregulation of anabolic markers (collagen type II) [85,109,111]. The enrichment of EVs with miR-140-5p significantly improved the level of osteochondral protection from OA progression [84], and gait abnormalities in the DMM model were found to be alleviated following exosomes treatment [85].

In studies using an osteochondral defect model, EV treatment resulted in complete neo-tissue infilling and the development of hyaline-like cartilage that was integrated with the surrounding tissue [107,112,113,114]. An increase in PCNA-presenting cells and decrease in CCP3 apoptotic cells was also observed, along with the switching of macrophage phenotype from M1 (pro inflammatory) to M2 (anti-inflammatory) and suppression of inflammatory cytokines [113]. In the study that used a cardiotoxin-induced muscular injury model, EVs were shown to accelerate muscular regeneration, and have an anti-inflammatory function that increased M2 anti-inflammatory markers and reduced M1 inflammatory markers [82].

## 4. Extracellular Vesicles from Mesenchymal Stem Cells for the Treatment of Rheumatoid Arthritis

Rheumatoid arthritis (RA) is the second most common form of arthritis, after OA. There is no cure for RA, with the only treatments being physical therapy and medication to help relieve symptoms and slow disease progression. RA is an autoimmune disease that is primarily defined by chronic joint inflammation, together with bone erosion and ECM destruction [116]. The pathogenesis of RA is driven by pre-existing genetic disposition coupled with risk factors that increase the likelihood of disease progression, such as smoking, silica dust exposure, vitamin D deficiency, and obesity [117]. The transition to a chronically inflamed synovium in RA is incompletely understood, although it may be triggered by a number of antagonistic stimuli such as local microtrauma, microvascular injury, and complement activation. Additionally, autoantibodies can activate periarticular osteoclasts, a step which initiates bone damage and is associated with the release of TNF-α and IL-8, both of which promote synovitis [118]. Once synovitis is established, the synovial ECM is disrupted, and there is activation of stromal cells together with a myriad of infiltrating cells including T-cells, B-cells, macrophages, mast cells, and plasma cells. Disease progression involves a complex and incompletely understood relationship between cells and soluble immune factors, most notably chemokines and cytokines [4].

Driven by the well-known anti-inflammatory and immunomodulatory functions of MSCs, a handful of studies have explored the use of MSC-derived EVs as a therapeutic for RA. In contrast to the number of publications available for OA, there were only five studies that applied MSC-derived EVs to experimental models of RA or inflammatory arthritis, as shown in Table 1. Of these, four studies sourced MSCs from the bone marrow [119,120,121,122], and one from RA synovial fluid and human neutrophils [123]. All studies applied exosomes as the EV subpopulation of interest, including one that used both exosomes and MVs [123]. The exosomes used in most studies were characterised to be approximately 100 nm in diameter [119,121,122], while the diameter of MVs was within the range of 150–575 nm [120].

For the in vitro experiments, two studies implemented a RA cell model comprising fibroblast-like synoviocytes (FLS) isolated from the diseased tissue of RA patients [119,121]. The invasion of RA FLS into cartilage and angiogenesis are important processes mediating the pathogenesis of RA. Both studies explored the enrichment of exosomes with a specific miRNA, namely miR-150-5p [119] or miR-124a [121]. Both studies showed that the miRNA-enriched exosomes could inhibit the increased migration and invasion associated with RA FLS. In one study, a co-culture of RA FLS with human umbilical vein endothelial cells (HUVECs) that was treated with miR-150-5p-enriched exosomes substantially inhibited tube formation, compared to exosomes that were enriched with a control miRNA (miR-67) [119], suggesting that the former could suppress angiogenesis. When treated with inflammatory cytokines, RA FLS were shown to upregulate MMP-14 and vascular endothelial growth factor (VEGF), but this upregulation was attenuated when the cells were treated with miR-150-5p-enriched exosomes [119]. These findings led to the hypothesis that miR-150-5p delivered by exosomes could be one of the mechanisms responsible for suppressing cell migration, invasion, and tube formation in RA. In the other study, the effects of miR-124a-enriched exosomes on RA FLS were compared with unaltered exosomes by evaluating proliferation, cell cycle progression, apoptosis, and ‘wound’ closure (modelled by a wound scratch assay) [121]. The miR-124a-enriched exosomes were found to induce a more pronounced suppression of proliferation, arrest of the cell cycle in the G0/G1 phase rather than the S phase, stronger inhibition of the wound closure healing rate, and similar levels of apoptosis in RA FLS compared to unaltered exosomes [121]. One other study involving an in vitro investigation explored the immunomodulatory effects of exosomes and MVs derived from both IFN-γ-primed and un-primed murine bone marrow MSCs on murine T- and B-lymphocytes [120]. Both primed and un-primed MSC culture media were found to suppress the proliferation of T-lymphocytes. After centrifugation, the culture medium supernatant lost its ability to suppress proliferation, suggesting that the immunomodulatory agents secreted by MSCs were present in the EV-containing pellet. The EVs were shown to have dose-dependent effects on suppressing T-lymphocyte proliferation, but these suppressive effects were lost when the EVs were subjected to a freeze-thaw cycle. Both exosomes and MVs were shown to have the ability to suppress several types of pro-inflammatory cells (CD8+ IFN-γ+ and CD4+ IFN- γ+ cells) and increase the number of anti-inflammatory cells (CD4+ IL-10+ Tr1 and CD4+ CD25+ Treg).

Three studies performed in vivo experiments using a collagen-induced arthritis (CIA) model in mice or rats. Two explored the enrichment of exosomes with specific miRNAs, miR-150-5p [119] or miR-192-5p [122]. The third used exosomes and MVs derived from IFN-γ-primed and un-primed murine bone marrow MSCs [120]. Treating CIA mice with miR-150-5p-enriched exosomes resulted in reduced levels of inflammation, as measured by hind paw thickness and reduced clinical arthritis scores compared to PBS and exosomes enriched with a control miRNA (miR-67) [119]. Mice treated with miR-150-5p-enriched exosomes also showed downregulation of VEGF (angiogenic factor), CD31 (angiogenesis marker), and MMP-14 (promotes the characteristic invasion of FLS into cartilage). When miR-192-5p-enriched exosomes were injected into CIA rats, they were found to migrate from the bloodstream into synovial tissue, where they downregulated the expression of a number of factors that normally show upregulation in the CIA model (RAC2, TRAP, PGE2, IL-1β, TNF-α, NO, and iNOS) and upregulated miR-192-5p expression [122]. To identify an optimal dose of EVs for the CIA model, a study comparing exosomes and MVs first employed a delayed type hypersensitivity (DTH) model to measure inflammation through paw swelling [120]. Both exosomes and MVs exerted a dose-dependent anti-inflammatory effect and were the most effective at the maximum dose of 250 ng. Carrying this dose into the CIA model, the exosomes-treated group developed arthritis at a rate of 5% with extremely low clinical arthritis scores, compared to the MVs-treated group at 20% and higher clinical arthritis scores. Both EV groups performed significantly better than the PBS-treated group, which developed arthritis at a rate of 47% with relatively severe clinical arthritis scores. In a separate experiment using the CIA model, a 250-ng dose of exosomes was shown to have a superior anti-inflammatory effect compared to 500 ng of MVs, as assessed through paw swelling, lower rates of arthritis development, and lower clinical arthritis scores [120]. In addition, one study used both K/BxN- and glucose-6-phosphate isomerase (GPI)-induced inflammatory arthritis models [123]. Treatment with a neutrophil-derived EV population that likely contained both exosomes and MVs was found to reduce cartilage degradation and proteoglycan loss, and this chondroprotective effect was shown to require AnxA1. The interactions of AnxA1 in MVs with the FPR2/ALX receptor was suggested to increase TGF-β production by chondrocytes.

## 5. Extracellular Vesicles from Mesenchymal Stem Cells for the Treatment of Alzheimer’s Disease

Alzheimer’s disease (AD) is the most common form of dementia, characterised by a progressive and irreversible degeneration of the central nervous system (CNS). The pathological hallmarks of AD include extracellular amyloid-β plaques, neurofibrillary tangles, and neuronal dysfunction and degeneration [124]. Chronic neuroinflammation is now broadly understood to play an important role in the pathogenesis of AD, which involves activated microglia and the release of numerous cytokines to produce a sustained immune response [125]. Due to its complex pathophysiology, there are currently no effective treatments for AD. The only approved drugs used for AD treatment provide only symptomatic relief rather than slowing the progression of disease.

Due to their anti-inflammatory, pro-regenerative, and immunomodulatory characteristics, MSCs have been recently considered as a possible treatment for AD [126]. However, despite the promising trophic functions of these cells demonstrated in preclinical models of AD, there are concerns surrounding their intracranial transplantation in a clinical setting, which is associated with increased risks of complications and mortality [126]. In this context, the use of MSC-derived EVs may provide a breakthrough strategy for the treatment of AD, that can convey the therapeutic effects of the parent cells while exhibiting the advantages of small size, low immunogenicity, and lack of ability to undergo malignant transformation.

As shown in Table 1, the 11 studies on AD demonstrated a range of therapeutic effects elicited by MSC-derived EVs in both in vitro and in vivo experimental models. The MSCs were derived from various sources, including bone marrow [127,128,129,130,131], adipose tissue [132], umbilical cord [133,134], Wharton’s Jelly [135], and commercially obtained MSCs with an unspecified origin [136]. One study also used neural stem cells (NSCs) isolated from the hippocampus of mice as the cell source [137]. Half of the studies used a heterogeneous population of EVs [128,129,130,132,135], while the other half used exosomes [127,131,133,134,136,137]. Different size ranges and protein markers were used to identify EV populations in these studies.

Six of the studies involved in vitro experiments, four of which used amyloid-β oligomers (AβO) to induce disease, since this has been identified as the major toxic species driving neuroinflammation in AD. Two studies used hippocampal cells [128,135], one used primary cortex neurons [132], and one used microglia [133] as AβO-induced AD models. The other two studies used an AD model comprising microglia exposed to inflammatory cytokines (TNF-α and IFN-γ) [130], or neuroblastoma cells transfected with amyloid precursor protein (APP) gene to overexpress Aβ peptides [134]. In the two studies that used hippocampal cells, one used neurons exclusively [128], while the other used a mixture of hippocampal cells including neurons and astrocytes, as well as a neuron-only oxidative stress model [135]. The hippocampal cells treated with AβO showed a much higher level of EV uptake than control cells, although it was also noted that the uptake of EVs was primarily performed by astrocytes rather than the neuronal cells [135]. The EVs were shown to have an anti-oxidant effect, as they protected against synapse damage [128] and prevented the formation of reactive oxygen species (ROS) in hippocampal cells induced by AβO [135]. However, once the catalase (a known anti-oxidant) content of EVs was inactivated, these protective effects of EVs were lost [128,135]. In the two studies that used microglia, one showed that, after AβO treatment, the application of exosomes increased the markers for alternative activation into the anti-inflammatory M2 microglia phenotype, as well as induced higher levels of anti-inflammatory cytokines (IL-10 and TGF-β) and lower levels of pro-inflammatory cytokines (IL-1β and TNF-α) [133]. The other study showed that, after microglial pre-treatment with inflammatory cytokines, the application of EVs significantly reduced the production of the pro-inflammatory cytokines IL-6 and IL-1β and increased the production of the anti-inflammatory cytokine IL-10 [130]. In the remaining AD cell models, the EVs were shown to reverse neuronal toxicity [132] and reduce the levels of secreted and intracellular Aβ [134].

From the nine studies that performed in vivo investigations, eight used transgenic mice pre-determined to develop an early onset of AD [127,129,130,131,132,133,134,137], and one used an amyloid-β aggregate-induced AD mouse model [136]. Treatment with EVs led to a range of beneficial effects, including reduced plaque deposition in both the cortex and hippocampus [127,129,131]; reduced levels of soluble and insoluble amyloid-β aggregates [127,129,132,133,134]; improved memory and spatial learning abilities as assessed by the Morris water maze (MWM) test [127,131,133,134,136,137]; improved memory as assessed by the Novel Object Recognition (NOR) test [132,136]; reduced levels of pro-inflammatory markers (including IL-6, IL-β, IL-α, IL-1β, and TNF-α) [127,133,137]; increased levels of anti-inflammatory markers (including IL-13, IL-10, IL-4, and TGF-β) [127,133]; increased levels of the AβO degrading enzymes neprilysin (NEP) [131,133] and insulin-degrading enzyme (IDE) [133]; reduced numbers of pro-inflammatory M1 microglia [130,133]; and increased numbers of anti-inflammatory M2 microglia [133]. One study explored the conjugation of EVs with the rabies virus glycoprotein (RVG) to improve targeting of the nerve tissue [127]. Compared to unconjugated EVs, conjugated EVs showed increased migration from the bloodstream into the cortex and hippocampus. This resulted in lower plaque deposition in these brain regions, as well as greater reduction in AβO aggregates, increased levels of anti-inflammatory markers, decreased levels of pro-inflammatory markers, and improved spatial learning and memory ability as assessed by MWM. Another interesting study cultured MSCs on graphene 2D films or 3D scaffolds and compared the effects of the resulting exosomes [134]. The 3D exosomes were found to be much more effective at improving spatial learning and memory, reducing Aβ deposition, and reducing neuroinflammation and oxidative stress in AD mice compared to 2D exosomes. These differences were thought to be related to the topographical structures of the 3D scaffold, which might have led to increased secretion of protective factors by the parent MSCs that were then captured in the exosomes.

## 6. Extracellular Vesicles from Mesenchymal Stem Cells for the Treatment of Cardio Vascular Disease

Acute myocardial infarction (AMI) is the leading cause of death in people with cardiovascular disease worldwide. AMI is characterised by insufficient blood supply to the heart leading to cardiac ischemia, decrease in functioning cardiomyocytes, and death of myocardial tissue [29,138]. The irreversible progression of cardiomyocyte and myocardial tissue necrosis negatively impact cardiac function and can result in congestive heart failure [29,138]. Following AMI, inflammatory cells including macrophages, monocytes, and neutrophils migrate to the infarcted myocardium to induce repair. However, the resulting inflammatory response may persist for longer than necessary and lead to further cardiac damage [139]. MSCs can potentially protect the myocardium following AMI by suppressing persistent inflammation, stimulating angiogenesis and the differentiation of fibroblasts within the infarcted region, abrogating apoptosis, and alleviating fibrosis, hence repairing the myocardium and likely preventing further cardiac dysfunction or heart failure [138].

Due to the anti-inflammatory, pro-angiogenic and anti-oxidant effects of MSCs, MSC-derived EVs have been recently investigated for their reparative role in cardiovascular disease [140,141]. The immunomodulatory role of MSC-derived exosomes was shown in an in vitro study that used MSCs from healthy human donor bone marrow [140]. The exosomes exhibited prominent anti-inflammatory properties on peripheral blood mononuclear cells (PBMCs), where they attenuated the pro-inflammatory factor TNF-α and elevated the anti-inflammatory factor TGF-β during in vitro culture. At the later stages of tissue repair, TGF-β is also pro-fibrotic with a well-established role in cardiac fibrosis, cardiomyocyte apoptosis, and cardiac hypertrophy through activin receptor-like kinase activity (ALK) [142]. The physiological process of cardiac repair post AMI consists of three major stages: inflammatory, proliferative, and maturation. Fibroblasts are the key ‘reparative’ cardiac cells during the inflammatory stage, which are capable of degrading ECM components and releasing inflammatory mediators (IL-1β, TNF-α) to enhance the inflammatory response during cardiac rupture [138]. In contrast, myofibroblasts exhibit an anti-inflammatory phenotype important for the maturation stage of cardiac repair by secreting ECM components and anti-inflammatory factors (TGF-β), and enhancing the differentiation of fibroblasts into myofibroblasts [138].

As seen in Table 1, the 14 included studies all showed therapeutic effects of MSC-derived EVs in various small animal models of cardiovascular disease. Exosomes from several MSC sources including bone marrow, adipose tissue, and umbilical cord were all shown to have effects in increasing angiogenesis and cardiac function in a left anterior descending (LAD) coronary artery ligation myocardial infarction (MI) rat model [29]. Related to the process of cardiac repair following AMI as described above, a study showed that exosomes derived from human umbilical cord MSCs can attenuate the inflammation induced by cardiac fibroblasts in vitro, and also have cardioprotective effects in vivo in a lipopolysaccharide (LPS)-induced rat model of MI [138]. These findings were supported by a similar study where PBS, MSCs, and MSC-derived exosomes were injected into the peri-infarct zone of rat infarcted myocardium, where the treatment group receiving exosomes showed the most prominent decrease in the infiltration of CD68+ inflammatory cells [141]. The MSC-derived exosomes were shown to exert their beneficial effects through different mechanisms compared to the parent MSCs. In an LAD coronary artery ligation MI rat model, MSCs and MSC-derived exosomes showed notable distinctions in the expression of several miRNAs [141]. For example, the expression levels of miR-21 and miR-15 were significantly lower in exosomes compared to in MSCs. The downregulation of these miRNAs in exosomes might act to suppress hypertrophy and reduce cardiac ischaemic injury, which might explain their greater therapeutic benefits compared to MSCs. Other mechanisms by which MSC-derived exosomes may reduce cardiomyocyte apoptosis in MI is by regulating cell autophagy through AMPK/mTOR and Akt/mTOR signalling [143].

Dilated cardiomyopathy (DCM) and inflammatory cardiomyopathy (ICM) are some of the most multifaceted complications of myocardial inflammation, characterised by ventricular enlargement and subsequent systolic dysfunction [144]. In a doxorubicin-induced model of dilated cardiomyopathy, cardiac dilation was ameliorated by MSC-derived exosomes, which also showed anti-inflammatory effects that decreased the expression of IL-1, IL-6, and TNF-α cytokines. In addition, these exosomes suppressed the pro-inflammatory macrophages (ILY6C^high^ and M1-like F4/80+ CD11c+) and elevated anti-inflammatory macrophages (LY6C^low^ and M2-like F4/80+ CD206+) in the blood and heart of the treated mice, which restored macrophage polarisation through activation of the JAK2-STAT6 pathway [144]. Similar effects were demonstrated in a clodronate liposome-induced myocardial ischaemia-reperfusion (I/R) injury model, where MSC-derived exosomes were capable of preserving cardioprotective efficacy, reducing inflammation of heart tissue, and inducting macrophage polarisation by shuttling the miR-182 gene to downregulate inflammatory toll like receptor 4 (TLR 4) and upregulate the pro-survival PI3k/Akt signalling pathway [145]. The immunomodulatory effects of MSC-derived exosomes were demonstrated by reduced M1 gene expression markers (iNOS, IL-1β, IL-6, TNF-α) and increased M2 gene expression markers (Arg1, IL-10, CD206, TGF-β) both in vitro and in vivo [145]. In a D-galactose aging-induced cardiac dysfunction model, exosomes derived from umbilical cord MSCs were shown to inhibit cardiac inflammation by increasing the expression of the long noncoding RNA (lncRNA) MALAT1, which suppressed NF-kB/TNF-α signalling pathways [146]. Rat bone marrow MSC-derived exosomes carrying miRNA-125b exhibited substantial therapeutic effects on a myocardial I/R injury rat model, leading to a reduction in inflammatory factors (IL-β, IL-6, and TNF-α) by downregulating the SIRT7 gene and thus improving heart function [147]. The miRNA-125b abrogated the infarct area and provided protection from myocardial ischemia reperfusion injury [147]. The exosomes derived from LPS-primed bone marrow-derived MSCs showed a potential role in macrophage polarisation and tissue repair in several inflammatory models [148]. 

In a mouse MI model, exosomes from bone marrow-derived MSCs, both primed or un-primed with LPS, were shown to reduce inflammation and myocardial injury by strongly attenuating LPS-dependent NF-κB signalling and activating the Akt1/Akt2 pathway [149]. The LPS-primed MSC-derived exosomes were found to have the greatest effects on cardiac inflammation, cell viability, and apoptosis. In rat cardiomyocytes and rats treated with doxorubicin and trastuzumab to induce oxidative stress, exosomes derived from human mesenchymal progenitor cells obtained from right cardiac appendage tissue were found to reduce cell death, and provide protection against cardiac dysfunction and myocardial fibrosis [150]. These exosomes were shown to be enriched in miR-146a-5p, which might act to suppress a number of target genes that encode signalling mediators of inflammation and cell death in cardiomyocytes.

To increase the efficacy of MSC-derived EVs, drug delivery systems have been developed to enable their intramyocardial delivery post-AMI. One approach is to encapsulate the EVs within a self-assembling peptide (SAP) hydrogel modified with a SDKP pattern [151]. The MSC-derived EVs were administered by intramyocardial injection in a rat MI model, either alone or in conjugation with an angiogenic and anti-inflammatory SAP hydrogel based on (RADA)_4_-SDKP [151]. This was found to increase α-smooth muscle actin (SMA) vessel-like structures to promote angiogenesis, as well as reduced inflammation and pro-inflammatory CD68+ cells, which resulted in improved ventricular remodelling and cardiac function. Similarly, exosomes encapsulated in a functional peptide hydrogel could be released in a prolonged and stable manner in a rat model of MI, resulting in reduced cardiac damage and improved angiogenesis [152]. In another study, lentivirus packaging was used to engineer exosomal-enriched membrane protein (Lamp2b) fused with ischemic myocardium-targeting peptide CSTSMLKAC (IMTP) [153]. The IMTP-exosomes produced by transfected MSCs were shown to induce a greater reduction in mRNA levels of pro-inflammatory factors (TNF-α, IL-6, and IL-1β) within the ischaemic heart area in an AMI model compared to blank exosomes. The group treated with IMTP-exosomes also showed a reduced percentage of M1 macrophages (TNF-α+ CD68+) and increased percentage of M2 macrophages (CD206+) compared to groups treated with blank exosomes or PBS. Furthermore, the IMTP-exosomes were found to reduce cardiac cell apoptosis and infarct size, and improve vasculogenesis. In an interesting study that combined the delivery of exosomes and MSCs in a rat MI model, MSC-derived exosomes were given through intramyocardial injection, with or without intravenous infusion of atorvastatin-pretreated MSCs on days 1, 3, or 7 after MI [154]. The combined treatment of exosomes followed by MSC transplantation was found to improve cardiac function, reduce infarct size, and increase neovascularisation to a greater extent than controls treated with exosomes or MSCs alone. Furthermore, the highest improvement in heart function was achieved by the group that received exosomes followed by MSCs at day 3 after MI. The delivery of exosomes before MSC transplantation was found to enhance cell survival and reduce cell apoptosis.

## 7. Extracellular Vesicles from Mesenchymal Stem Cells for the Treatment of Preeclampsia

Preeclampsia is a life-threatening cardiovascular disorder occurring in the second-half of pregnancy with multifactorial pathogenesis [155,156]. Although a leading cause of maternal and neonatal morbidity and mortality, there are no current treatments for preeclampsia. The exact pathogenic mechanisms of preeclampsia are not well understood, but two phenotypes have been identified based on the time of diagnosis: early-onset (before 34 weeks of gestation) and late-onset (from 34 weeks of gestation) [155,156]. Early onset preeclampsia is often associated with inappropriate placentation due to inadequate trophoblast invasion leading to placental hypoxia. Late-onset preeclampsia is characterised by irregular growth of the placenta and underlying maternal cardiovascular, metabolic, and inflammatory conditions [156,157,158]. Nevertheless, both phenotypes display inflammation, which is often mediated by abnormally upregulated immune responses and activation of the innate immune system and pro-inflammatory factors [158]. Imbalance between ROS and anti-oxidants due to placental hypoxia can also lead to systemic inflammation and endothelial dysfunction before preeclampsia is manifested. The pathogenic mechanisms underlying these processes appear to involve the secretion of syncytiotrophoblast macrovesicles, inflammatory factors such as TNF-α, and anti-angiogenic factors such as soluble fms-like tyrosine kinase (sFlt-1) and Endoglin (sEng) into the maternal circulation [156].

MSCs have gained growing attention as a potential therapeutic strategy to alleviate inflammation, oxidative stress, and restricted angiogenesis in preeclampsia [156]. MSC treatment has been shown to cause deactivation of both innate and adaptive inflammatory immune cells including monocytes, macrophages, dendritic cells (DCs), CD4+ and CD8+ cells, natural killer (NK) cells, and B-cells, as well as activation of a regulatory sub-set of immune cells to resolve inflammation in preeclampsia [156]. MSC-derived EVs have recently been explored as an alternative, safer and more effective option to whole cell-based therapy for the treatment of preeclampsia. A number of studies have investigated the functional role of the miRNA cargo of MSC-derived EVs, which can metabolically induce changes in macrophage phenotype from a glycolysis-conducted M1 pro-inflammatory phenotype to an oxidative phosphorylation-dependent M2 anti-inflammatory phenotype [156].

From the four studies shown in Table 1 on testing MSC-derived EVs in models of preeclampsia, two were in vitro studies performed using HUVECs [159] or trophoblasts [160] as cell models, while the other two were in vivo studies performed in rat models of preeclampsia [161,162]. EVs derived from human decidua MSCs were found to enhance HUVEC proliferation by reducing inflammation and oxidative stress [159]. The addition of EVs to LPS-treated HUVECs also caused a significant reduction in pro-inflammatory IL-6 cytokine concentration. On the other hand, exosomes from human umbilical cord MSCs were found to promote cell proliferation, migration and cell cycle entry, as well as inhibit apoptosis in human trophoblast cell lines [160]. These effects were increased when exosomes were derived from the same MSCs transfected with miR-133b. The exosomal miR-133b was shown to exert its effects on trophoblasts by regulating glucocorticoid-regulated kinase 1 (SGK1).

The two studies that performed in vivo testing to investigate the effects of MSC-derived EVs in rat models of preeclampsia both used human umbilical cord MSCs as the cell source for producing EVs. One study using exosomes from the MSCs showed that these significantly lowered blood pressure and proteinuria in the animal model, both key features of preeclampsia [161]. From in vitro experiments performed in the same study, it was noted that the exosomes could promote trophoblast cell migration, invasion and proliferation while reducing apoptosis, possibly by transferring miR-139-5p to the trophoblasts and thereby regulating the ERK/MMP-2 pathway. In the other study, the exosomes were administered at low, medium or high doses to the animals [162]. Their effects included lowering blood pressure and proteinuria, as well as increasing the number and quality of foetuses, quality of the placenta, microvascular density of the placenta, and VEGF expression while decreasing cell apoptosis and the expression of the anti-angiogenic factor sFlt1. It was interesting to note that the effects of exosomes were exerted in a dose-dependent manner.

## 8. Limitations and Future Perspectives of Using Extracellular Vesicles from Mesenchymal Stem Cells for the Treatment of Inflammation-Related Conditions

As demonstrated by the studies discussed in this review, the use of MSC-derived EVs has potential to provide substantial therapeutic benefits in a range of inflammation-related conditions, including osteoarthritis, rheumatoid arthritis, Alzheimer’s disease, cardiovascular disease, and preeclampsia. To date, a small number of studies have been published on applying MSC-derived EVs for each of these conditions in experiments involving in vitro and small animal models of disease. The field of using MSC-derived EVs as therapeutics is still in its infancy, but holds significant promise as a viable option in the management and treatment of inflammatory conditions. Compared to injecting live cells, MSC-derived EVs offer a prominent advantage in the ability to be used as off-the-shelf therapeutics, since they can be preserved in cold storage for long periods of time without loss of biological activity upon thawing, for example, at −20 °C for six months [163] or at −80 °C for up to two years [164]. The isolation and purification of EVs from the conditioned medium that has been used to culture MSCs is expected to be conducted under sterile conditions, removing the need to perform additional sterilisation prior to EV application, unlike for locally administered pharmacological agents. Although comprehensive analyses are still emerging in the field, preclinical studies have demonstrated that EVs have minimal toxicity and immunogenicity, even when applied xenogenetically as a large dose at high frequency [89]. There is a potential safety concern with intravascular injection of large quantities of EVs, particularly MVs that are shed from the cell membrane, if they express the same levels of prothrombotic tissue factor (TF/CD142) and collagen type-1 (COL-1) as the parent MSCs, which may trigger coagulation pathways and lead to blood clots [165]. This is an area that warrants further investigation, although it is less concerning in the local delivery of MSC-derived EVs to specific tissues, which is the typical method of administration for regenerative medicine applications. Although MSC-derived EVs show promising characteristics and preclinical evidence, there remain a number of considerable questions to answer and limitations to address before the application of these therapeutic EVs can be translated to a clinical setting.

The exact mechanisms underlying the therapeutic effects of EVs when applied to different diseases remain incompletely understood in current studies, posing an area where significantly more work is needed. In particular, the optimal tissue source of MSCs for generating EVs has been rarely explored. A wide variability in therapeutic efficacy has been seen across the many MSC sources used in different studies, including bone marrow, adipose tissue, synovial membrane, induced pluripotent stem cells (iPSCs), and embryonic stem cells (ESCs). Direct comparison between studies is difficult due to significant variations in the culture media and conditions used to generate EVs, isolation methods, experimental set-up, and EV dosage. Only one study so far has directly compared two different MSC sources, which showed that EVs derived from iPSCs had superior therapeutic efficacy for the treatment of OA compared to those derived from synovial membrane MSCs [110]. In addition, few studies have investigated the influence of environmental conditions and stresses on the molecular cargo and effectiveness of EVs generated by stem cells [34,166]. These observations raise multiple important questions: (i) which MSC source possesses the greatest therapeutic potential for a given condition, (ii) which factors are critical in determining the therapeutic potential of EVs, and (iii) how to optimise the therapeutic potential of EVs for a chosen MSC source. The posing of these open questions is further supported by interesting observations from a study showing that EVs from adipose-derived MSCs cultured in hypoxic conditions exerted a greater regenerative effect than those cultured in normoxic conditions [82], as well as others demonstrating that the therapeutic potential of MSC-derived EVs could be improved through enrichment with specific miRNAs [83,84,85,119,121,145,148]. In addition to the cell source, another aspect of EV-based therapy that has not been sufficiently explored is the dosage and frequency of treatment. Studies which compared variable dose levels of EVs all tend to demonstrate the best therapeutic outcomes in the highest dose group, creating difficulties in finding an ‘optimal’ level of EV administration. There is also a wide variation in treatment frequency among the studies included in this review. Further investigations are needed to elucidate the most effective treatment method for a given disease that takes into consideration the source of MSCs, dose of EVs and frequency of administration, among other important factors.

The currently available evidence on the therapeutic applications of MSC-derived EVs in inflammatory conditions are limited to in vitro and small animal studies exploring the short-term outcomes of EV treatment. There is a critical need for future studies to investigate the long-term effects. A question remains about whether the regenerative outcomes and disease attenuation induced by EVs are permanent or temporary. It is possible that the short-term positive outcomes of EV treatment observed in current studies are a result of temporarily altered gene expression, and that given sufficient time the diseased phenotype may return. Addressing this question will enable researchers to determine if EV treatment will require ongoing administration, and if so, the frequency that is required to produce a sustained therapeutic effect. Furthermore, it is crucial that research on EVs as therapeutics is translated to large animal studies, using models such as sheep or pigs that may more closely mirror disease progression and treatment response in humans.

For EVs to become a widely accepted and applied treatment option, a number of technical limitations pertaining to the general field of EV research needs to be addressed. Currently, there is no consensus on the optimal methods for EV generation, isolation, purification and characterisation. To upscale the technology surrounding the use of EVs as therapeutics, standardisation of these processes is essential [167,168]. EV isolation is currently achieved through techniques such as centrifugation, immunoaffinity isolation, polymeric precipitation, size exclusion, and microfluidic devices [47]. Unfortunately, all of these methods carry certain limitations which reduce the ability to upscale the EV production, such as insufficient exclusion capacity resulting in contamination, alteration of EV structure resulting in a loss of function, and poor isolation capacity resulting in incomplete EV fraction isolation and low yield [169]. Another significant challenge lies in the generation of a sufficient quantity of EVs for practical applications, such as for preclinical animal models or clinical trials. The current yield of EVs from MSCs in conventional culture conditions is extremely low, with 1 L of culture medium conditioned with a total of approximately 60 million MSCs only producing 1–2 mg (protein content) of EVs [170]. With single injections in mice of up to 500 μg EVs used in some studies [104] and many others conducting weekly injections, it remains a considerable hurdle to upscale these EV quantities to clinically viable treatment options for humans.

One method that has been explored in the hope of increasing the therapeutic efficacy of EVs is to load them within a hydrogel for delivery. When delivered in an aqueous solution (typically saline), EVs are either rapidly absorbed by the tissue at the site of injection or dissipate to other areas, thereby reducing their effectiveness. The rationale for developing EV-loaded hydrogel delivery systems is to enable the sustained release of EVs over an extended period of time. The feasibility of this approach has been reported in a number of studies, where MSC-derived EVs exhibited increased therapeutic potential to treat chronic liver failure in a rat model [171] or renal I/R injury in a mouse model [172], when delivered in vivo within a polymerised hydrogel compared to EVs delivered in an aqueous medium.

The characterisation and quantification of EVs is another area lacking consensus in the field [167,168]. EV quantification is commonly performed through dynamic light scattering or nanoparticle tracking analysis, the characterisation of surface markers through protein analysis by flow cytometry or western blotting, and morphological analysis through electron microscopy [173]. Nevertheless, there remains significant variation among studies regarding the types of methods selected for EV characterisation. In addition, the nomenclature for the classification of EVs is currently not consistently defined across the literature, with different studies implementing varying size ranges and protein markers for distinguishing different EV subpopulations such as exosomes and MVs. The importance of having a standardised set of methods and definitions cannot be overstated, as it is a requirement for clinical application that EVs can be produced in a consistent and accurate manner to ensure that maximum therapeutic potential along with safety outcomes are consistently achieved.

The current research strongly suggests that MSC-derived EVs have potential to provide significant anti-inflammatory and regenerative effects on damaged or diseased tissues in inflammation-related conditions, particularly when locally administered in large doses. While the landscape for using MSC-derived EVs in the future as a new generation of therapeutics appears promising, there is a critical need for enhancing the efficacy and robustness of EVs, and standardising the methods of EV production and isolation.

## Figures and Tables

**Table 1:** Current evidence of the therapeutic effects of EVs in a range of inflammation-related conditions.

**OSTEOARTHRITIS**					
**Study**	**Source of EVs**	**Type of EVs**	**Administration and Dose**	**Model(s) Used**	**Major Findings**
Cosenza et al., 2017 [104]	Mouse bone marrow-derived MSCs	Exosomes (112 ± 6.6 nm) and MVs (223 ± 14.5 nm)	In vitro: Exosomes and MVs were applied to cells at 12.5 ng, 125 ng, or 1.25 μg In vivo: Intra-articular injection of 2.5 × 10^5^ MSCs, 500 ng MVs or 250 ng exosomes in 5 μL saline, at seven days after OA induction	In vitro: Mouse chondrocytes treated with IL-1β (1 ng/mL) In vivo: Collagenase-induced mouse model of OA; analysis at 42 days after OA induction	In vitro: - Exosomes and MVs enhanced anabolic marker expression and decreased catabolic marker expression; at high doses, the effects of EVs on cells were similar to those treated with MSCs - Inhibited macrophage activation In vivo: - Exosomes and MVs both showed chondroprotective effects, presenting with healthy articular cartilage that had no discernible difference compared to healthy controls at 42 days - Improved subchondral bone quality and partially prevented ligament and meniscus calcification
Li et al., 2020 [105]	Mouse bone marrow-derived MSCs	Exosomes (120.31 ± 15.28 nm)	EVs (200 μg exosomes in 200 μL PBS) were injected weekly immediately after surgery for four weeks through the tail vein of mice	In vivo: Lumbar facet joint resection-induced mouse model of OA	- EVs had a powerful analgesic effect - Attenuated cartilage degeneration - Induced higher proteoglycan levels, and downregulation of MMP-13 and ACAN - Caused regeneration of osteochondral tissue, as well as maintenance and regeneration of subchondral bone - Blocked angiogenesis and aberrant nerve invasion
Liu et al., 2018 [115]	Human MSCs (from ATCC)	Exosomes (100 nm)	In vitro: Cells treated with 1, 5, or 10 µg/mL exosomes for 24 h In vivo: Intra-articular injection of exosomes from untreated MSCs and MSCs with knockdown of the lncRNA KLF3-AS1, at 21 days after OA induction	In vitro: Rat chondrocytes treated with IL-1β In vivo: Collagenase-induced rat model of OA; analysis at eight weeks	In vitro: - Partially reversed the changes induced by IL-1β treatment of cells, including reduced expression of COL2A1 and aggrecan, increased expression of MMP-13 and RUNX2, reduced migration and proliferation, and increased apoptosis In vivo: - Partially reversed cartilage degradation and expression of OA-indicative genes - Exosomes with lncRNA KLF3-AS1 knockdown had the opposite effect, suggesting that exosomal KLF3-AS1 could promote cartilage repair and chondrocyte proliferation
Liu et al., 2017 [114]	Human iPSC-derived MSCs	Exosomes (50–150 nm)	Exosomes were integrated into a hydrogel in a 20 μL volume at 1 × 10^11^ particles/mL, or given by intra-articular injection directly in the same volume and concentration	In vitro: Human chondrocytes or bone marrow-derived MSCs In vivo: Full-thickness osteochondral defects (4 mm diameter, 3 mm depth) in rabbits	In vitro: - Increased the migration of chondrocytes and MSCs - When cells were hydrogel encapsulated, exposure to exosomes improved their viability In vivo: - Exosomes suspended in the hydrogel formed in situ and integrated well with surrounding cartilage - 7 days after implantation, treated joints showed superior cell infiltration including chondrocytes, inflammatory cells, fibroblasts, and blood cells - 12 weeks after implantation, treated joints showed complete defect filling with smooth hyaline-like cartilage (high levels of organised collagen type II and low levels of collagen type I), and complete integration with native tissue
Lo Sicco et al., 2017 [82]	Human adipose-derived MSCs cultured in normoxic (20% O_2_) or hypoxic (1% O_2_) atmosphere	Mostly exosomes but also contained MVs (4–250 nm)	Intramuscular injection of 1 μg EVs in 20 μL PBS, injected 2 h after muscular injury and repeated after four days in muscular injury model	In vitro: Mouse bone marrow-derived macrophages In vivo: Mouse cardiotoxin-induced muscular injury model (analysis at 1, 2, and 7 days after injury)	In vitro: - MSCs cultured in normoxic or hypoxic conditions produced EVs with the same size and morphology - Both EV groups increased the expression of angiogenic molecules and induced epithelial tube formation, with hypoxic EVs being more angiogenic - Both EV groups were internalised by macrophages and increased macrophage proliferation; both demonstrated anti-inflammatory properties and ability to switch macrophages from M1 to M2 phenotype; hypoxic EVs had greater effects - Hypoxic EVs showed downregulation of 48 miRNAs and upregulation of 20 miRNAs; 4 of the upregulated miRNAs were associated with inflammatory processes (miR-223, miR-146b), proliferation and differentiation (miR-126, miR-199a) In vivo: - Both EV groups showed anti-inflammatory properties, with increase in M2 anti-inflammatory markers and decrease in M1 pro-inflammatory markers - Both EV groups showed accelerated regeneration of muscle tissue, although hypoxic EVs had a greater effect
Mao et al., 2018 [83]	Human bone marrow-derived MSCs	Exosomes (50–150 nm)	Intra-articular injection of exosomes from untreated MSCs and MSCs overexpressing miR-92a-3p; 15 μL at 500 μg/mL injected at 7, 14, and 21 days after OA induction	In vitro: Human chondrocytes treated with IL-1β and human MSCs undergoing chondrogenesis In vivo: Collagenase-induced rat model of OA; analysis at 28 days	In vitro: - Enhanced chondrocyte proliferation and migration - Upregulated COL2A1, SOX9 and aggrecan, and downregulated MMP-13, WNT5A, COL10A1 and RUNX2 in MSCs undergoing chondrogenesis In vivo: - Exosomes overexpressing miR-92a-3p reversed the OA-related changes (reduced COL2A1 and aggrecan in the cartilage matrix) to levels slightly below normal cartilage tissue, suggesting that they can reverse cartilage degradation in OA
Ragni et al., 2019 [108]	Human adipose-derived MSCs	Not specified; both small and large EVs	EVs were added to cells at a ratio of 100,000:1 (EVs:cell) for 10 days, with EVs freshly supplemented every two days	In vitro: Inflammatory OA cell model, comprising human FLSs isolated from OA patients and stimulated with IL-1β at physiological levels (25 pg/mL)	- Downregulated HAS3, MMP1, MMP13, CCL2 and CCL5 gene expression, and significantly (50%) downregulated CXCL8 expression from 2 to 10 days - EVs showed clear anti-inflammatory properties, possibly mediated by direct interaction with hyaluronan matrix and miRNA release
Tao et al., 2017 [84]	Human synovial membrane-derived MSCs	Exosomes (30–150 nm)	Intra-articular injection of exosomes from untreated MSCs and MSCs overexpressing miR-140-5p; 100 μL at 1 × 10^11^ particles/mL injected weekly from the fifth to eighth week after OA induction	In vitro: Human articular chondrocytes In vivo: Rat OA model induced through medial collateral ligament and medial meniscus transection; analysis at 12 weeks	In vitro: - Exosomes with normal miR-140-5p content increased chondrocyte proliferation and migration, but decreased ECM secretion levels - Exosomes with upregulated miR-140-5p increased chondrocyte proliferation and migration while maintaining ECM secretion levels In vivo: - Exosome with normal miR-140-5p content were mildly chondroprotective - Exosomes with upregulated miR-140-5p had much stronger chondroprotective abilities and the treated joint showed a very low level of osteochondral degeneration
Vonk et al., 2018 [106]	Human bone marrow-derived MSCs	Exosomes (40–150 nm)	EVs were added to cells over a period of 48 h (inflammatory model) or 28 days with repeated treatment every five days (cartilage regeneration model)	In vitro: Inflammatory model–human OA chondrocytes stimulated with TNF-α; cartilage regeneration model–human OA chondrocytes cultured in fibrin matrices	- In inflammatory model, EVs suppressed TNF-α and COX2 expression, as well as pro-inflammatory interleukins (IL-1α, IL-1β, IL-6, IL-8, IL-17) - In cartilage regeneration model, EVs improved proteoglycan content and upregulated the production of collagen type II
Wang et al., 2017 [111]	Human ESC-derived MSCs	Exosomes (38–169 nm)	Intra-articular injection of 1 × 10^6^ particles in 5 μL PBS in the bilateral joints, every 3 days for 28 days after OA induction	In vitro: Mouse articular chondrocytes treated with IL-1β In vivo: DMM mouse model of OA	In vitro: - Reversed the increased synthesis of collagen type II and ADAMTS5 expression in IL-1β-treated chondrocytes In vivo: - Significant chondroprotection and attenuation of OA progression - Maintained collagen type II content and lower levels of ADAMTS5
Woo et al., 2020 [109]	Human adipose- derived stem cells	Not specified; mostly small EVs (around 100 nm)	In vitro: Cells treated with 1 × 10^8^ or 2 × 10^8^ particles/mL In vivo: Intra-articular injection of 1 × 10^8^ particles in a 30 μL volume per joint	In vitro: Human OA chondrocytes treated with IL-1β In vivo: MIA rat model of OA and DMM mouse model of OA	In vitro: - Reduced catabolic gene expression - Reduced MMP-1, MMP-3, MMP-13, and ADAMTS5 expression - Increased collagen type II synthesis - Reduced expression of pro-inflammatory and cartilage degradation genes In vivo: - Reduced cartilage erosion, OA progression, and proteoglycan degradation - Reduced synovial inflammation and inhibited M1 macrophage infiltration into the synovium - Reduced MMP-13 expression - Maintained adipocyte content of infrapatellar fat pad - Did not demonstrate subintimal fibrosis and neovascularisation
Wu et al., 2019 [85]	MSCs from human intrapatellar fat pad	Exosomes (average 121.9 nm)	In vitro: Cells treated with different concentrations (1, 5, or 10 × 10^8^ particles/mL) in the presence or absence of IL-1β (10 ng/mL) In vivo: Intra-articular injection of 1 × 10^10^ particles/mL in 10μL twice a week for four or six weeks	In vitro: Human OA chondrocytes In vivo: DMM mouse model of OA	In vitro: - Increased chondrocyte viability and cell migration - Partially reversed IL-1β-induced apoptosis - Reversed IL-1β-induced changes to ADAMTS5, MMP-13 and collagen type II expression In vivo: - Attenuated cartilage degradation and promoted cartilage maintenance - Reversed DMM-induced changes to MMP-14, ADAMTS5, and collagen type II expression
Xiang et al., 2018 [107]	Human bone marrow-derived MSCs	MVs (average 200 nm)	In vitro: Cells were treated with 5–20 μL MVs isolated from 1 × 10^6^ MSCs, for 12 h In vivo: Intra-articular injection of 30 μL MVs at three days after defect creation	In vitro: Human chondrocytes treated with IL-1β (10 ng/mL) In vivo: Osteochondral defects (4 mm diameter, 5 mm depth) in the medial femoral condyle of rabbits	In vitro: - Cells showed EV uptake, which was CD44 dependent - Increased proliferation potential and decreased apoptosis rate In vivo: - At 20, 40, and 60 days, MV-treated group caused gradual defect filling with hyaline-like tissue rich in collagen type II, reaching the heigh of native cartilage; PBS-treated group showed no significant regeneration, with adipose and fibrotic cells progressively filling the defect
Zhang et al., 2018 [113]	MSCs derived from a human embryonic cell line	Exosomes (average 100 nm)	Intra-articular injection of 100 μg exosomes in 100 μL PBS once per week for 12 weeks after OA induction	In vitro: Rat chondrocytes In vivo: Osteochondral defects (1.5 mm diameter, 1 mm depth) in the knee joint of rats; analysis at 2, 6, and 12 weeks	In vitro: - Cells showed increased migration and proliferation potential proportional to the dose of exosomes - Increased ECM synthesis and decreased apoptosis In vivo: - Promoted hyaline-like cartilage regeneration - Increased collagen type II and s-GAG content - Increased cell proliferation and apoptosis attenuation - Caused increase in cells presenting PCNA and a more rapid decrease in CP3 apoptotic cells - Increased abundance of M2 macrophages in the synovium and cartilage, and decreased M1 macrophages and inflammatory cytokines
Zhang et al., 2016 [112]	MSCs derived from a human embryonic cell line	Exosomes (average 100 nm)	Intra-articular injection of 100 μg exosomes in 100 μL PBS once per week for 12 weeks after OA induction	In vivo: Osteochondral defects (1.5 mm diameter, 1 mm depth) in the knee joint of rats; analysis at 6 and 12 weeks	- Attenuated cartilage degeneration - Caused regeneration of hyaline cartilage, and increased GAG and collagen type II content - Caused complete regeneration of subchondral bone
Zhu et al., 2017 [110]	Human synovial membrane-derived MSCs, and human iPSC-derived MSCs	Exosomes (50–150 nm)	Intra-articular injection of 1 × 10^10^ particles/mL in 8 μL PBS at 7, 14, and 21 days after OA induction	In vitro: Human articular chondrocytes In vivo: Collagenase-induced mouse model of OA; analysis at 28 days	In vitro: - Both exosome groups improved chondrocyte migration and proliferation, with exosomes from iPSC-derived MSC showing more dramatic improvements In vivo: - Both exosome groups showed significant improvements in reducing OA pathology compared to controls - Exosomes from iPSC-derived MSCs showed a greater regenerative potential, presenting with smooth hyaline-like cartilage, normal collagen type II localisation and healthy proteoglycan content
**RHEUMATOID ARTHRITIS**					
**Study**	**Source of EVs**	**Type of EVs**	**Administration and Dose**	**Model(s) Used**	**Major Findings**
Chen et al., 2018 [119]	Mouse bone marrow-derived MSCs	Exosomes (average 100 nm)	Intradermal injection of 50 μg exosomes in 100 μL PBS twice per week; exosomes were from MSCs overexpressing miR-150-5p or the control miR-67	In vitro: FLSs isolated from RA patients, and HUVEC and FLS co-culture In vivo: CIA mouse model	In vitro: - Exosomes enriched with miR-150-5p substantially inhibited tube formation in HUVEC-FLS co-culture, as well as migration and invasion of RA FLSs, indicating that they can suppress angiogenesis - miR-150-5p from enriched exosomes suppressed the expression of MMP-14 and VEGF, but miR-67 did not have the same effects In vivo: - Exosomes enriched with miR-150-5p induced downregulation of MMP-14 and VEGF in tissue, reduction in the thickness of the hind paw, and lower clinical scores of arthritis compared to groups treated with miR-67 control exosomes or PBS - Substantially improved arthritis severity and successfully inhibited angiogenesis
Cosenza et al., 2018 [120]	Mouse bone marrow-derived MSCs	EVs separated into exosomes (average 120 nm, expressing CD81 and CD 9) and MVs (150–600 nm, expressing Sca-1, CD44 and CD29)	Intravenous injection of 250 ng exosomes or 600 ng MVs in CIA mouse model at 18 and 24 days after arthritis induction	In vitro: Mouse spleen T- and B-lymphocytes In vivo: Mouse delayed T hypersensitivity (DTH) model (injection into footpad at five days after immunisation; analysis at 24 h after injection), and CIA mouse model (analysis at 30 days)	In vitro: - EVs from MSCs primed with IFN-γ showed a dose-dependent effect on T-lymphocyte suppression, but their immunomodulatory effects were lost after freeze-thawing - Primed and un-primed exosomes and MVs suppressed ConA-activated splenocytes to a similar extent - MSCs exerted the strongest suppression of CD8+ IFN-γ+ cells, followed by similar levels by exosomes and MVs - MSCs, exosomes and MVS all increased the percentage of CD4+ IL-10+ Tr1 cells - Exosomes and MVs both increased the percentage of CD4+ CD25+ Treg cells, while MSCs had no effect - Isolated CD4+ and CD8+ cells treated with exosomes or MVs did not show reduced proliferation In vivo: - In DTH model, both exosomes and MVs showed anti-inflammatory effects that were dose-dependent - In CIA model, exosomes and MVs showed significant protection from developing arthritis; treatment with exosomes showed 5% rate of developing arthritis, and this 5% had very low clinical arthritis scores; treatment with MVs did not exert significant protection from arthritis symptoms, but reduced the incidence to 20% with low clinical scores - Exosomes and MVs both showed maintenance of subchondral bone, with exosomes being more effective than MVs
Headland et al., 2015 [123]	Human RA synovial fluid, and human neutrophils (stimulated or not stimulated with TNF-α)	MVs (0.05–1 μm; may contain both exosomes and MVs)	Intra-articular injection of 3 × 10^4^ particles in 5 μL PBS in k/BxN-induced model at 3 days after arthritis induction, or in GPI-induced model at 21 days after arthritis induction	In vitro: Human chondrocyte micromass, and ex vivo rat cartilage explant In vivo: Inflammatory arthritis rat models induced by k/BxN (analysis at five days after induction) or GPI (analysis at 25 days after induction)	In vitro: - Protected against cartilage degradation by reducing IL-8 and PGE2 release, ECM degradation, and chondrocyte apoptosis - EVs from neutrophils showed the ability to migrate through the ECM in rat cartilage explants, and migration was increased in explants treated with IL-1β; EVs needed to remain intact to migrate and exert chondroprotective effects - Although neutrophil-derived EVs had chondroprotective effects, direct contact between neutrophils and chondrocytes induced apoptosis In vivo: - Neutrophils injected into damaged joints migrated towards zones of inflammation where they released EVs, which showed the ability to penetrate cartilage ECM - Protective effects of neutrophil-derived EVs were thought to be related to AnxA1 and FPR2/ALX interactions, which increased the production of TGF-β in chondrocytes
Meng et al., 2020 [121]	Human bone marrow-derived MSCs	Exosomes (approximately 100 nm)	Cells were treated with 20 μg/mL exosomes from MSCs overexpressing miR-124a	In vitro: Human RA FLS cell line	- Exosomes enriched with miR-124a showed the ability to enter cells, providing significant numbers of exosomes and high levels of miR-124a in cells - Suppressed RA FLS proliferation, inhibited wound closure healing rate at 24 h, and inhibited migration and invasion - Exosomes enriched with miR-124a arrested the cell cycle in the G0/G1 phase, compared to control exosomes at the S phase - Both miR-124a-enriched and control exosomes promoted RA FLS apoptosis
Zheng et al., 2020 [122]	Rat bone marrow-derived MSCs	Exosomes (101 ± 14.45 nm)	Injection of 50 μg exosomes in 100 μL PBS twice per week, starting at three weeks after the second arthritis induction procedure; exosomes were from MSCs overexpressing miR-192-5p	In vivo: CIA rat model, with two inductions 21 days apart	- Exosomes enriched with miR-192-5p showed the ability to migrate from the bloodstream to synovial tissues, where they significantly increased miR-192-5p expression and reduced RAC2 expression - Reduced TRAP activity (usually elevated in CIA model) - Significantly attenuated the elevated levels of PGE2, IL-1β, and TNF-α levels in synovial tissues, and levels of NO and iNOS in serum (usually elevated in CIA model); exosomes enriched with miR-192-5p had greater effects than control exosomes
**ALZHEIMER’S DISEASE**					
**Study**	**Source of EVs**	**Type of EVs**	**Administration and Dose**	**Model(s) Used**	**Major Findings**
Bodart-Santos et al., 2019 [135]	Human Wharton’s jelly mesenchymal stem cells	Not specified, contained both exosomes and MVs (mostly 50–300 nm)	Cells were cultured with 6 × 10^8^ EV particles	In vitro: Hippocampal cells were isolated from hippocampi from 18-day old rat embryos, and conditioned or not with AβOs for 2 h	- Control cells showed low uptake of EVs, while AβO-treated cells showed much higher levels of EV uptake - EV uptake was primarily performed by astrocytes rather than neuronal cells - Once catalase in EVs was inactivated, the EVs no longer prevented the formation of ROS in AβO-treated cells - EV treatment of AβO-treated cells for 22 h prevented synapse damage
Cui et al., 2019 [127]	Mouse bone marrow-derived MSCs	Exosomes, tagged with rabies viral glycoprotein (RVG) which targets the CNS	Intravenous injection of tagged and untagged exosomes at 5 × 10^11^ particles in 100 μL PBS, once per month for four months	In vivo: APP/PS1 transgenic mouse (prone to early onset of AD)	- Conjugated RVG-exosomes travelled from the bloodstream to the cortex and hippocampus at much greater numbers than unconjugated exosomes - Conjugated exosomes greatly reduced the levels of cortex and hippocampus plaque deposition, soluble Aβ40 and Aβ42, insoluble Aβ40 and Aβ42, and GFAP expression (astrocyte marker) in the brain; unconjugated exosomes showed smaller effects - Conjugated exosomes caused improved spatial recognition and memory retention as shown through MWM test - Conjugated exosomes substantially downregulated the pro-inflammatory markers IL-1α, IL-1β and IL-6, and upregulated the anti-inflammatory markers IL-4, IL-10 and IL-12
de Godoy, 2018 [128]	Rat bone marrow-derived MSCs	Not specified, contained both exosomes and MVs (mostly 50–300 nm)	Cells were cultured with 8 × 10^7^ EV particles (corresponds to ~5000 MSCs), dose tripled in some experiments	In vitro: Hippocampal neuronal cells treated with AβOs	- Prevented AβO-induced synapse damage in neurons - EVs that had catalase (an antioxidant) removed lost the ability to protect against oxidative stress - Functional ability of EVs was maintained after cryopreservation
Ding et al., 2018 [133]	Human umbilical cord-derived MSCs	Exosomes (30–150 nm)	In vitro: Cells were cultured with exosomes at 30 μg/mL In vivo: Intravenous injection of 30 μg exosomes in 100 μL PBS every two weeks for eight weeks	In vitro: Mouse BV2 microglia cell line In vivo: APP/PS1 transgenic mouse	In vitro: - Cells showed alternative activation into anti-inflammatory M2 microglia, with decreased levels of pro-inflammatory cytokines (IL1β, TNF-α) and increased levels of anti-inflammatory cytokines (IL-10, TGF-β) In vivo: - Increased memory as shown through MWM test - Decreased numbers of Aβ plaques in the hippocampus and cortex, and soluble Aβ40 and Aβ42 in the brain - Greatly increased the levels of NEP and IDE (Aβ-degrading enzymes) - Decreased levels of Iba-1 positive (pro-inflammatory M1) microglia, and increased expression of markers for anti-inflammatory M2 microglia
Elia et al., 2019 [129]	Mouse bone marrow-derived MSCs	Not specified, contained both exosomes and MVs (mostly 50–300 nm)	Intracerebral injection of 22.4 μg EVs (1 × 10^9^ particles) in 4 μL PBS	In vivo: APP/PS1 transgenic mouse, two age groups (three and five months; earliest signs of cognitive impairment appear at six months); analysis at 25 days after injection	- Reduced Aβ plaque area and density in the hippocampus and cortex - Reduced plaque solidarity in the neocortex (site of injection) - Lower numbers of plaques surrounded by dystrophic neurites - Introduces the possibility for intervention before clinical manifestation of AD
Li et al., 2020 [137]	Mouse hippocampus NSCs	Not specified (50–190 nm)	Injection of 200 μg EVs in 10 μL PBS bilaterally into the lateral ventricles, twice a week for four weeks	In vivo: APP/PS1 transgenic mouse	- Increased memory as shown through MWM test - Upregulated the expression of mitochondrial function-related factors and synaptic proteins - Significantly reduced the ratio of damaged to total synapses - Significantly reduced the levels of pro-inflammatory IL-1β, IL-4, IL-10, p65, and TNF-α, as well as Iba-1 expression compared to vehicle control - No significant differences in the levels of soluble and insoluble Aβ40 and Aβ42 between EV-treated and vehicle control groups
Losurdo et al., 2020 [130]	Human bone marrow-derived MSCs	Not specified, contained both exosomes and MVs (average 200 nm)	In vitro: Cells were cultured with EVs (4.5 μg/mL) from MSCs pre-conditioned with TNF-α and IFN-γ In vivo: Intranasal spurts of 5 μL EV solution (300 μg/mL) totalling 100 μL, given twice at 18 h apart	In vitro: Microglia cultures consisting of hippocampal and cortical astrocytes, treated with TNF-α and IFN-γ In vivo: Female triple-transgenic AD mouse expressing three mutant human transgenes (PS1M146V, APPSwe, and tauP301L)	In vitro: - Reduced expression of pro-inflammatory markers IL-6 and IL-1β, and increased expression of the anti-inflammatory marker IL-10 - Completely or partly attenuated the negative effects exerted by pro-inflammatory cytokine treatment In vivo: - Reduced Iba-1+ cell density, together with reduction in microglial cell body size - Reduced CD68 expression associated with the activated microglia phenotype
Ma et al., 2020 [132]	Human adipose MSCs	EVs (130 ± 28 nm)	In vitro: Cells were cultured with EVs for 24 h In vivo: Intranasal administration of 10 μL EVs at the protein dose of 1 mg/kg every two days for two weeks	In vitro: Primary neurons from embryonic mice, treated with Aβ1-42 oligomers or glutamate In vivo: APP/PS1 transgenic mouse	In vitro: - RNA sequencing showed neuroprotective effects of EVs, with some upregulated genes important for synaptic function, and downregulated genes related to cell death - Significantly reversed neuronal toxicity induced by Aβ1-42 oligomers or glutamate; increased cell viability In vivo: - Reduced neurologic damage in whole brain areas, and remarkably increased neurogenesis - Slightly reduced Aβ deposition and microglia activation - Rescued memory deficits as shown through NOR and Y-maze tests
Reza-Zaldivar et al., 2019 [136]	MSCs (from ATCC)	Exosomes (size unspecified)	Intra-peritoneal injection of 10 μg exosomes in 2 μL PBS; analysis at 14 and 28 days after injection	In vivo: C57BL/6 mouse treated with Aβ aggregates, and AD allowed to develop for 14 days before intervention	- Improved spatial learning and memory as shown through MWM test - Increased scores in NOR test - Stimulated expression of neurogenesis markers in the subventricular zone - Increased numbers of immunoreactive cells compared to PBS control, but similar numbers compared to MSC-treated group
Wang et al., 2020 [131]	Mouse bone marrow-derived MSCs	Exosomes (approximately 110 nm)	Injection of exosomes (50 μg in 80 μL saline) through the cauda vein, every two weeks for 16 weeks	In vivo: APP/PS1 transgenic mouse	- Significantly improved spatial learning and memory ability as shown through MWM test - Reduced amyloid levels in the cortex and hippocampus, and enhanced the expression of NeuN - Reduced Aβ1-40, Aβ1-42, BACE1, and PS1 expression, and promoted NEP expression in the brain - Effects were mediated by activating the SphK/S1P signalling pathway
Yang et al., 2020 [134]	Human umbilical cord MSCs	Exosomes (50–150 nm)	In vitro: Cells were cultured with 2 μg exosomes for 24 h; exosomes were isolated from MSCs cultured on 2D graphene film or 3D graphene scaffold In vivo: Exosomes were delivered by infusion into the right hippocampus, at 0.25 μL/h (2 mg protein/mL) for 14 days	In vitro: AD pathology cell model, comprising SH-SY5Y cells transfected with amyloid precursor protein (APP) gene, leading to increased production of Aβ peptides In vivo: APP/PS1 transgenic mouse	In vitro: - 3D-exosomes had greater effect in upregulating α-secretase and downregulating β-secretase to reduce levels of secreted and intracellular Aβ In vivo: - 3D exosomes were more effective at improving spatial learning and memory function, as shown through MWM test - Exosomes were mainly concentrated at the site of delivery but also distributed throughout the brain parenchyma; 3D exosomes were more effective at decreasing Aβ deposition by eliminating production and facilitating clearance of Aβ - Exosomes reduced neuroinflammation by attenuating microglial activation, and markedly reduced oxidative stress; 3D exosomes produced more pronounced effects
**CARDIOVASCULAR DISEASE**					
**Study**	**Source of EVs**	**Type of EVs**	**Administration and Dose**	**Model(s) Used**	**Major Findings**
Chen et al., 2020 [147]	Rat bone marrow MSCs	Exosomes (60–100 nm), enriched with miR-125b or control miR-67	Exosomes (50 μg) were injected into the ligation zone contiguous to the left anterior free wall after left ventricle exposure	In vivo: Rat I/R model, in which the LAD was ligated for 30 min followed by reperfusion for 2 h	- Increased cell viability and inhibited inflammation, oxidative stress and apoptosis - Reduced infarct size and improved cardiac function, with increased LVEF, LVFS and LVSP - Upregulated miR-125b and anti-apoptotic Bcl-2, and downregulated pro-apoptotic factors Bax and caspase-3 - Decreased the levels of inflammatory factors IL-1β, IL-6 and TNF-α - Downregulated SIRT7 gene and protein expression
Firoozi et al., 2020 [151]	Human bone marrow MSCs	EVs (average 100 nm)	EVs (80 μg) were injected in a 100 μL volume, directly or encapsulated in an SAP hydrogel, into four sites of the infarct border zone after ligation	In vitro: Neonatal mouse cardiomyocytes treated with H_2_O_2_ In vivo: LAD coronary artery ligation rat model of MI	In vitro: - Protected cardiomyocytes from damage due to H_2_O_2_-induced oxidative stress In vivo: - Improved LVEF and LVFS, promoted cardiac morphological status and preserved function - Reduced fibrosis area, apoptosis and inflammation; reduced expression of pro-inflammatory CD68+ cells - Promoted angiogenesis in infarcted myocardium, with increased numbers of α-SMA+ structures - Both encapsulated and free EVs improved cardiac function, likely by reducing macrophage infiltration and increasing angiogenesis
Han et al., 2019 [152]	Human umbilical cord MSCs	Exosomes (size unspecified)	Exosomes (20 μg) were injected in a 20 μL volume, directly or encapsulated in a peptide hydrogel, into two different sites next to the infarcted border region after ligation	In vitro: Rat H9C2 cardiomyoblasts treated with H_2_O_2_ In vivo: LAD coronary artery ligation rat model of MI	In vitro: - Exosomes protected cell damage from H_2_O_2_-induced oxidative stress and improved cell viability In vivo: - Improved cardiac function with increased LVEF and LVFS - Reduced infarct size and length, fibrosis area, and apoptosis - Promoted angiogenesis in infarcted myocardium - Hydrogel facilitated prolonged and stable release of exosomes in the area of ischaemic myocardium - Decreased expression of TGF-β1 (pro-fibrosis), CD68 (indicative of inflammatory cell infiltration), and TNF-α (pro-inflammatory) - Increased diameter of CD31+ blood vessels and reduced number of apoptotic cells - Growth hormone releasing peptide-6 (GHRP6) was released following hydrogel degradation, activating pro-survival PI3K/Akt1 and TGF-β1 pathways and inhibiting the NF-kB pathway
Huang et al., 2019 [154]	Rat bone marrow MSCs	Exosomes (average 100 nm)	Exosomes (10 μg in 100 μL PBS) were injected at 3 sites around the infarct border 30 min after ligation, with or without intravenous delivery of atorvastatin-pretreated MSCs through the tail vein at day 1, 3, or 7 after MI	In vivo: LAD coronary artery ligation rat model of MI	- Exosome and MSC combinatorial treatment caused improved cardiac function with increased LVES and LVFS, reduced infarct size and collagen area, and increased neovascularisation (microvascular density in both arteriolar and capillary vessels) compared to exosomes or MSCs alone - Intramyocardial injection of exosomes 30 min after AMI combined with MSC transplantation at 3 days after AMI achieved the highest improvement in heart function - Combinatorial therapy reduced inflammation, with increased expression of the pro-survival protein Bcl-2, reduced number of apoptotic cells, and decreased levels of inflammatory cytokines IL-6 and TNF-α
Liu et al., 2017 [143]	Rat bone marrow-derived MSCs	Exosomes (50–100 nm)	In vitro: Cells were cultured with exosomes (10 µg/mL) for 6, 12, and 24 h In vivo: Exosomes (5 μg in 10 μL PBS) were injected into the anterior and lateral part of the visibly injured region 5 min before reperfusion	In vitro: Rat H9C2 cardiomyoblasts treated with H_2_O_2_ In vivo: Rat I/R model, in which the LAD was ligated for 30 min followed by reperfusion for 2 h	In vitro: - Enhanced cell viability, and reduced cell apoptosis and ROS production after H_2_O_2_ stimulation - Increased cell autophagy, which was regulated by AMPK/mTOR and Akt/mTOR signalling; upregulated p-AMPK/AMPK ratio and downregulated p-Akt/Akt and p-mTOR/mTOR ratio In vivo: - Reduced cardiomyocyte apoptosis and infarct size - Improved cardiac function with increased LVEF and LVFS
Milano et al., 2020 [150]	Human cardiac-resident mesenchymal progenitor cells (obtained from right cardiac appendage tissue)	Exosomes (mostly < 150 nm)	In vitro: Cells were pre-treated with exosomes for 1 h before inducing oxidative stress In vivo: Exosomes (3 × 10^10^ particles in 100 μL) were injected into the tail vein at 5, 11, and 19 days after study commencement	In vitro: Neonatal rat ventricular myocytes treated with doxorubicin (Dox) and trastuzumab (Trz) to induce oxidative stress In vivo: Rats injected with six doses of Dox (days 1–11) and six doses of Trz (days 19–28)	In vitro: - Prevented increase in ROS induced by Dox/Trz - Enriched in miR-146a-5p compared to exosomes from human dermal fibroblasts; suppressed expression of *Traf6, Smad4, Irak1, Nox4,* and *Mpo* (known target genes of miR-146a-5p) in Dox-treated cells, which might provide protection against Dox-induced cell death In vivo: - Prevented Dox/Trz effects on left ventricular dysfunction, myocardial fibrosis, CD68+ macrophage infiltration, and iNOS expression
Shao et al., 2017 [141]	Rat bone marrow-derived MSCs	Exosomes (size unspecified)	In vitro: Cells were cultured with exosomes for up to 48 h In vivo: Exosomes (20 μg in 20 μL PBS) were injected at two different sites beside the infarct border region after ligation	In vitro: Rat H9C2 cardiomyoblasts or BJ fibroblasts treated with TGF-β In vivo: LAD coronary artery ligation rat model of MI	In vitro: - Enhanced proliferation capacity and inhibited apoptosis in H9C2 cells - Reduced TGF-β-induced α-SMA expression and inhibited fibroblast transformation - Compared to MSCs, exosomes had lower expression of miR-21 and miR-15 - Upregulated PI3k-Akt and mTOR pathways In vivo: - Decreased infiltration of CD68+ inflammatory cells, and inhibited apoptosis - Improved cardiac function with increased LVEF and LVFS
Shi et al., 2019 [138]	Human umbilical cord MSCs	Exosomes (mostly 100 nm)	Exosomes (400 μg) were given by intramyocardial administration during surgery	In vitro: Rat neonatal cardiomyocytes, and cardiac fibroblasts treated with LPS In vivo: LAD coronary artery ligation rat model of MI	In vitro: - Increased myofibroblast density and improved collagen contraction - Promoted fibroblast-to-myofibroblast differentiation in inflammatory environments - Reduced cardiomyocyte apoptosis - Decreased fibroblast migration, but no effect on fibroblast proliferation - Decreased expression of IL-1β and TNF-α, and increased expression of TGF-β In vivo: - Suppressed inflammatory response and improved cardioprotective effects
Sun et al., 2018 [144]	Mouse bone marrow MSCs	Exosomes (average 35 nm)	Exosomes (300 μg in 200 μL PBS) were injected intravenously through tail vein seven days after disease induction	In vivo: Doxorubicin-induced mouse model of dilated cardiomyopathy	- Improved cardiac function with increased LVEF and LVFS - Attenuated cardiac dilation and reduced cardiomyocytes apoptosis - Decreased expression of pro-apoptotic protein Bax, and increased expression of pro-survival protein Bcl-2 - Decreased levels of inflammatory cytokines IL-1, IL-6 and TNF-α in serum - Reduced pro-inflammatory ILY6C^high^ and M1-like F4/80+ CD11c+ macrophages, and elevated anti-inflammatory LY6C^low^ and M2-like F4/80+ CD206+ macrophages - Regulated macrophage polarisation through activation of the JAK2-STAT6 pathway
Wang et al., 2018 [153]	Mouse bone marrow MSCs	Exosomes (30–150 nm), engineered through lentiviral packaging technology	Exosomes (4 × 10^9^ particles or 50 μg) in 100 μL were injected intravenously through the tail vein after ligation	In vitro: Hypoxia-induced rat H9C2 cardiomyoblasts In vivo: LAD coronary artery ligation mouse model of MI	In vitro: - IMTP-exosomes produced by transfected MSCs were internalised to a greater extent by hypoxia-injured H9C2 cells than blank exosomes In vivo: - IMTP-exosomes allowed prolonged delivery in the region of ischaemic myocardium - Decreased inflammation and apoptosis, with reduced expression of pro-inflammatory factors (IL-6, TNF-α, IL-1β) - Reduced M1 macrophages (TNF-α+, CD68+) and increased M2 macrophages (CD206+) - Improved revascularisation and cardiac function, with increased capillary density and number of arterioles
Xu et al., 2020 [29]	Human MSCs from bone marrow, adipose tissue and umbilical cord	Exosomes (mostly < 100 nm) for all MSC types	Injection of exosomes or MSCs (1.5 × 10^6^ cells) in 150 μL PBS at the margin area of MI 30 min after ligation	In vivo: LAD coronary artery ligation rat model of MI	- Exosomes promoted angiogenesis, reduced infarct size, inhibited cardiomyocyte apoptosis, and improved microvascular density - Decreased LVESD and LVEDD, increased LVEF and LVFS, and improved cardiac function - Increased the levels of angiogenesis factors VEGF, bFGF, and HGF - Decreased adverse cardiac remodelling
Xu et al., 2019 [149]	Rat bone marrow-derived MSCs	Exosomes (mostly 100 nm)	Exosomes (from 1 × 10^6^ LPS-primed or non-primed MSCs) were injected at four sites into myocardium around the infarct border zone	In vitro: Rat peritoneal macrophages In vivo: LAD coronary artery ligation mouse model of MI	In vitro: - Exosomes decreased the expression of pro-inflammatory TNF-α, IL-6 and IL-1β, and increased the expression of anti-inflammatory IL-10; LPS-primed exosomes showed greater downregulation of pro-inflammatory cytokines - LPS-primed exosomes significantly reduced pro-inflammatory M1 macrophage protein markers (CD11b, iNOS) and elevated anti-inflammatory M2 macrophage protein markers (CD206, ArgI) - LPS-primed exosomes regulated macrophage polarisation by suppressing NF-κB signalling, and activating AKT1/AKT2 signalling In vivo: - Attenuated post-infarction inflammation by mediating macrophage polarisation, reduced cardiomyocyte apoptosis, and improved cardiac function; LPS-primed exosomes had greater effects
Zhao et al., 2019 [145]	Mouse bone marrow MSCs	Exosomes (50–150 nm)	Exosomes (50 μg in 25 μL PBS) were injected at three different points of the peri-infarct myocardial region after reperfusion	In vivo: Mouse I/R model, where the LAD was ligated for 45 min followed by reperfusion; for macrophage depletion studies, mice were intravenously injected with 150 μL (5 mg/mL) clodronate liposomes	- Improved cardiac function; reduced infarct size, fibrosis, hypertrophy of cardiomyocytes, and myocardial injury - Reduced infiltration of inflammatory cells and inflammation of heart tissue - Decreased pro-inflammatory cytokines (IL-6) and increased anti-inflammatory cytokines (IL-10) in serum and heart tissues - Reduced M1 macrophages and M1 gene expression markers (iNOS, IL-1β, IL-6, TNF-α), and increased M2 macrophages and M2 gene expression markers (Arg1, IL-10, CD206, TGF-β) - Downregulated TLR4, and upregulated PI3k/Akt signalling pathway through miR-182 in association with immunomodulation effects
Zhu et al., 2019 [146]	Human umbilical cord MSCs	Exosomes (size unspecified)	Exosomes (100 μg/injection) were injected in the tail vein three times per week for six weeks	In vitro: Rat H9C2 cardiomyoblasts treated with treated with H_2_O_2_ In vivo: Mouse model of aging and cardiac dysfunction induced by D-galactose	In vitro: - Promoted cell proliferation and prevented senescence - Inhibited the activity of the cell senescence mediator NF-κB and the expression of its subunit p-p65 - Exosomes prevented cell senescence through the lncRNA MALAT1, by inhibiting the NF-κB/TNF-α pathway In vivo: - Improved cardiac function with increased LVEF and LVFS - Attenuated the effects of D-galactose and preserved telomere length - Increased the anti-aging marker TERT and decreased the aging marker p21 - Reduced the expression of pro-inflammatory TNF-α - Protective effects of exosomes were blocked by silencing the lncRNA MALAT1
**PREECLAMPSIA**					
**Study**	**Source of EVs**	**Type of EVs**	**Administration and Dose**	**Model(s) Used**	**Major Findings**
Liu et al., 2020 [161]	Human umbilical cord MSCs	Exosomes (30–120 nm)	Abdominal injection of exosomes (160 μg/mL) at 0.5 mL/day from day 14 to 19 of gestation	In vitro: HTR-8/SVneo human trophoblast cell line In vivo: Rat preeclampsia model induced by L-NAME	In vitro: - Hindered cell apoptosis by inhibiting c-caspase-3 activity - Increased miR-139-5p expression in trophoblasts, showing pro-angiogenic and anti-inflammatory function - Inhibited PTEN expression and promoted ERK/MMP-2 pathway activation through miR-139-5p In vivo: - Decreased blood pressure and proteinuria - Restored miR-139-3p expression in placental tissue, and reduced expression of PTEN and MMP-2
Wang et al., 2020 [160]	Human umbilical cord MSCs	Exosomes (average 130 nm)	Cells were treated with exosomes for three days; exosomes were from MSCs overexpressing miR-133b	In vitro: HPT-8 and HTR8-S/Vneo human trophoblast cell lines	- Promoted trophoblast proliferation, migration, invasion ability, and cell cycle progression, and inhibited cell apoptosis; effects were more pronounced in exosomes enriched with miR-133b overexpression - In placental tissue of preeclampsia patients, miR-133b is downregulated and SGK1 is up-regulated - Exosomal miR-133b may act by reducing SGK1 expression
Xiong et al., 2018 [162]	Human umbilical cord MSCs	Exosomes (30–100 nm)	Abdominal injection of low (120 µg/mL), medium (140 µg/mL), or high (160 µg/mL) dose exosomes at 0.5 mL/day for 6 days, starting on day 14 of gestation	In vivo: Rat preeclampsia model induced by L-NAME	- Decreased blood pressure and proteinuria - Dose-dependent increase in foetus number and quality, as well as microvascular density - Decreased expression of anti-angiogenic sFlt1 and increased expression of pro-angiogenic VEGF in placental tissue, with the high exosome dose showing the most prominent effects
Zheng et al., 2020 [159]	Decidual MSCs from human placenta	Not specified, mostly small EVs (224.2 ± 4.2 nm)	Cells were treated with EVs (100 μg/mL, pooled from 6 patient samples) in 100 μL culture medium	In vitro: HUVECs treated with LPS or human preeclampsia serum	- Increased cell attachment and proliferation, and reduced production of pro-inflammatory IL-6 in both HUVEC models - No significant effect on lipid peroxidation in LPS-treated HUVECs, but significantly reduced lipid peroxidation in preeclampsia serum-treated HUVECs

AβO: amyloid-β oligomer; AD: Alzheimer’s disease; ATCC: American Type Culture Collection; bFGF: basic fibroblast growth factor; CIA: collagen-induced arthritis; CNS: central nervous system; DMM: destabilisation of the medial meniscus; ECM: extracellular matrix; ESC: embryonic stem cell; EV: extracellular vesicle; FLS: fibroblast-like synoviocytes; GAG: glycosaminoglycan; GPI: glucose-6-phosphate isomerase; HGF: hepatocyte growth factor; HUVEC: human umbilical vein endothelial cell; I/R: ischaemia-reperfusion; IDE: insulin-degrading enzyme; IFN: interferon; IL: interleukin; IMTP: ischemic myocardium-targeting peptide; iNOS: inducible nitric oxide synthase; iPSC: induced pluripotent stem cell; LAD: left anterior descending; lncRNA: long noncoding RNA; LPS: lipopolysaccharide; LVEF: left ventricular ejection fraction; LVFS: left ventricular fractional shortening; LVSP: left ventricular systolic pressure; LVESD: left ventricular end systolic diameter; LVEDD: left ventricular end diastolic diameter; MI: myocardial infarction; MIA: monosodium iodoacetate; MMP: matrix metalloproteinase; MSC: mesenchymal stem cell; MV: microvesicle; MWM: Morris water maze; NEP: neprilysin; NO: nitric oxide; NOR: Novel Object Recognition; NSC: neural stem cell; OA: osteoarthritis; PGE2: prostaglandin E2; PBS: phosphate buffered saline; PCNA: proliferating cell nuclear antigen; RA: rheumatoid arthritis; ROS: reactive oxygen species; s-GAG: sulfated glycosaminoglycan; SAP: self-assembling peptide; TGF: transforming growth factor; TNF: tumour necrosis factor; TRAP: tartrate-resistant acid phosphatase; VEGF: vascular endothelial growth factor.

## Abbreviations

AβO	amyloid-β oligomer
AD	Alzheimer’s disease
ATCC	American Type Culture Collection
bFGF	basic fibroblast growth factor
CIA	collagen-induced arthritis
CNS	central nervous system
DMM	destabilisation of the medial meniscus
ECM	extracellular matrix
ESC	embryonic stem cell
EV	extracellular vesicle
FLS	fibroblast-like synoviocyte
GAG	glycosaminoglycan
GPI	glucose-6-phosphate isomerase
HGF	hepatocyte growth factor
HUVEC	human umbilical vein endothelial cell
I/R	ischaemia-reperfusion
IDE	insulin-degrading enzyme
IFN	interferon
IL	interleukin
IMTP	ischemic myocardium-targeting peptide
iNOS	inducible nitric oxide synthase
iPSC	induced pluripotent stem cell
LAD	left anterior descending
lncRNA	long noncoding RNA
LPS	lipopolysaccharide
LVEF	left ventricular ejection fraction
LVFS	left ventricular fractional shortening
LVSP	left ventricular systolic pressure
LVESD	left ventricular end systolic diameter
LVEDD	left ventricular end diastolic diameter
MI	myocardial infarction
MIA	monosodium iodoacetate
MMP	matrix metalloproteinase
MSC	mesenchymal stromal cell
MV	microvesicle
MWM	Morris water maze
NEP	neprilysin
NO	nitric oxide
NOR	Novel Object Recognition
NSC	neural stem cell
OA	osteoarthritis
PGE2	prostaglandin E2
PBS	phosphate buffered saline
PCNA	proliferating cell nuclear antigen
RA	rheumatoid arthritis
ROS	reactive oxygen species
s-GAG	sulfated glycosaminoglycan
SAP	self-assembling peptide
TGF	transforming growth factor
TNF	tumour necrosis factor
TRAP	tartrate-resistant acid phosphatase
VEGF	vascular endothelial growth factor
